# Evolution of spatial structure, passing network patterns, and gameplay intensity in elite women’s and men’s football (2020–2025)

**DOI:** 10.1038/s41598-026-52701-6

**Published:** 2026-05-14

**Authors:** Rebecca Carstens, Raj Deshpande, Pau Esteve, Nicoló Fidelibus, Sara Linde Neven, Ramona Ottow, Lokamruth K.R., Paula Rodríguez-Sánchez, Luca Santagata, Javier M. Buldú, Brennan Klein, Maddalena Torricelli

**Affiliations:** 1 Roslin Institute, University of Edinburgh, Edinburgh, UK; 2 NPLab, Network Science Institute, Northeastern University London, London, UK; 3 Instituto de Física Interdisciplinar y Sistemas Complejos IFISC, Campus UIB, Palma de Mallorca, Spain; 4 Department of Science, Technology and Society, University School for Advanced Studies IUSS Pavia, Pavia, Italy; 5 Institute for Biodiversity and Ecosystem Dynamics, University of Amsterdam, Amsterdam, Netherlands; 6 Universitat Pompeu Fabra, Barcelona, Spain; 7 Department of Computer Science, Reykjavík University, Reykjavík, Iceland; 8 Complex Systems Group & GISC, Universidad Rey Juan Carlos, Móstoles, Madrid, Spain; 9 Department of Information Engineering and Computer Science, University of Trento, Povo, Italy; 10 Network Science Institute, Northeastern University, Boston, Massachusetts USA; 11 Department of Physics, Northeastern University, Boston, Massachusetts USA; 12 Institute for Experiential AI, Boston, Massachusetts USA

**Keywords:** Mathematics and computing, Physics, Psychology, Psychology

## Abstract

Elite football is believed to have evolved in recent years, yet systematic evidence for the pace and form of that change remains sparse. Drawing on event-level records for 13,018 matches across ten top-tier men’s and women’s leagues in England, Spain, Germany, Italy, and the United States (2020–2025), we quantify match dynamics through two complementary lenses: conventional performance statistics and pitch-passing networks that track ball movement across spatial regions of the field. Between 2020 and 2025, average passing volume, pass accuracy, and the proportion of passes made under pressure all increased, with the largest year-on-year changes occurring in women’s competitions. Network measures reveal that normalized outreach decreased, indicating teams increasingly concentrate ball circulation into shorter-range passing connections rather than wide spatial distribution. These trends are consistent across countries and tiers, yet persistent national differences indicate that stylistic diversity remains. Notably, women’s competitions exhibit stronger rates of change across most metrics, consistent with an accelerating professionalization, while the systematic decline in network outreach across all competitions is consistent with a sport-wide shift toward shorter, more concentrated passing structures.

## Introduction

Football has undergone profound transformations throughout its history, shaped by rule changes, technological advances, and shifting social contexts. The sport’s professionalization began in the late 19th century with the establishment of the English Football League in 1888^1^, yet women’s football faced decades of marginalization—including a ban on women’s matches at affiliated pitches in England from 1921 to 1971^2^. In recent years, the landscape has become increasingly dynamic: new teams join top-tier leagues; competitions expand and restructure; technological innovations such as the Video Assistant Referee alter gameplay; and women’s leagues experience unprecedented growth in visibility, investment, and institutional support^3,4^. This convergence of structural shifts, regulatory evolution, and cultural change makes contemporary football an inherently complex and rapidly evolving system.

The abundance of detailed match data in recent years has fueled the growth of sports analytics as a vibrant interdisciplinary field. Network science has been applied to analyze dynamics in various sports—including football^5^, basketball^6^, boxing^7^, badminton^8^, rugby^9^, and water polo^10^—with football being the most extensively studied. Different network representations have been employed to describe connections between players, teams, or leagues^11–17^. Match outcome data have been used to construct club networks assessing competitive influence^18^; event data have enabled identification of key players and analysis of player interactions^19–21^; and tracking data have supported studies of player coordination at fine spatial scales^22,23^. Advanced machine-learning methods now enable in-depth match analysis^24–28^, while research on tactical evolution documents changing playing styles across leagues^29–33^. Recent work has also examined the temporal dynamics of goal scoring and momentum effects^34^.

Despite this rich body of work, systematic comparative analyses tracking the evolution of tactical and strategic aspects of gameplay across both men’s and women’s competitions remain sparse. National football cultures shape how the game is played^32^: Spain is known for technical, possession-heavy football; Germany emphasizes structured organization and high pressing; Italy prioritizes tactical discipline and defensive solidity; England blends physicality with speed; and the United States focuses on athleticism and direct attacking play. Structural differences reinforce these tendencies: the English Premier League is highly commercialized with foreign ownership^35^; Spain and Italy also allow foreign ownership but with variable financial stability^36^; Germany’s Bundesliga enforces the “50+1” rule ensuring fan influence^37^; while Major League Soccer follows a franchise-based model with centralized ownership and no promotion/relegation^38^. Women’s leagues in these countries often share governance structures with their male counterparts^39^, yet playing styles are less distinctly defined due to ongoing professionalization and greater fluidity in player movement and coaching approaches^40^. Beyond structural and cultural differences, football’s evolution is shaped by season-to-season variability driven by player transfers, coaching changes, and external events. Major tournaments such as the 2022 FIFA World Cup and 2024 European Championship impact subsequent season starts as players require recovery time, affecting early-season performance patterns^41^. Within seasons, short-term dynamics including player fatigue, injuries, and team form influence match-level metrics. These temporal factors introduce complexity into evolutionary trend analysis, as improvements in some metrics may reflect adaptation to coaching systems, while declines in others may signal accumulated physical strain.

In this study, we investigate the evolution of football gameplay from a tactical and strategic perspective, analyzing men’s and women’s elite competitions in parallel across recent seasons. Our central question is: How have key performance indicators evolved across top-tier leagues between 2020 and 2025, and do these changes differ systematically across countries and between men’s and women’s football?

We analyze 13,018 matches from ten competitions across five countries (England, Spain, Germany, Italy, and the United States) spanning 2020 to 2025^42^. Our approach integrates two complementary lenses. First, we employ statistical analysis of team-level match performance indicators—such as passing volume, accuracy, and pressure—to quantify observable changes in gameplay. Second, we construct pitch-passing networks where spatial zones of the pitch serve as nodes connected by passes, allowing us to capture ball possession dynamics and spatial control^43–47^. By focusing on team-level attributes rather than individual players, we capture broader structural trends in playing style and match behavior.

Our analysis reveals systematic patterns in how football is played and how these patterns have changed over the past five seasons. We find increases in possession intensity and passes made under defensive pressure, particularly pronounced in European leagues, with women’s competitions exhibiting stronger rates of change across most metrics. Spatial organization shows divergent trends: declining network outreach and increasing vertical play suggest a shift toward more compact, direct attacking patterns. Country-level differences persist throughout, with Spain maintaining distinct tactical signatures in both offside rates and spatial distribution. Finally, we identify league-specific evolutionary trajectories—most notably in Italy’s Serie A Women, which shows distinct patterns of change compared to other competitions.

## Results

**Fig. 1:**
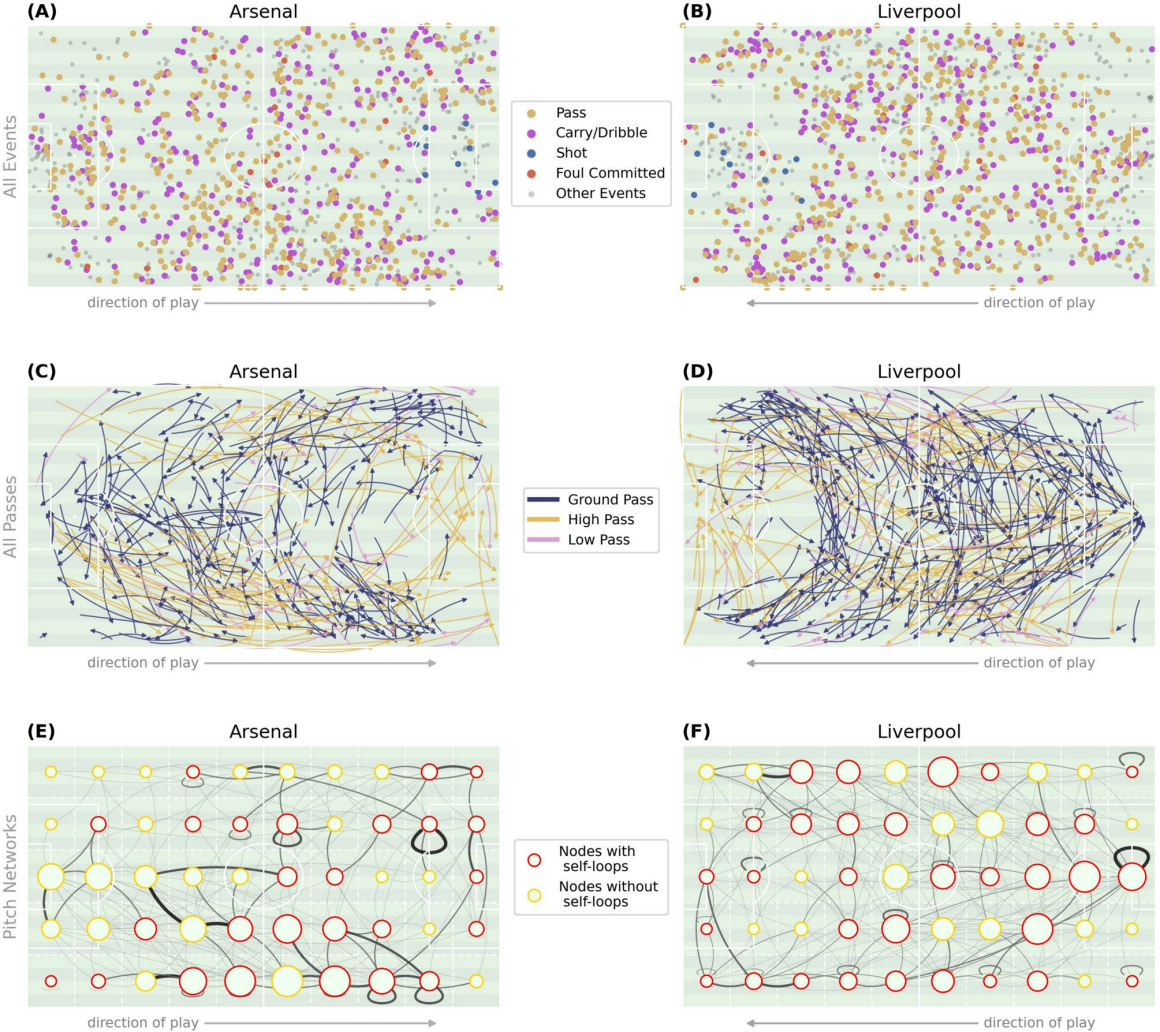
Pitch-passing network construction. Schematic illustration of the transformation from raw match events to pitch-passing networks, shown for a representative match between Arsenal and Liverpool. **(A,B)** All recorded events including passes (beige), carries/dribbles (purple), shots (blue), fouls (red), and other actions (gray). **(C,D)** Spatial distribution of passes, color-coded by type: ground passes (blue), high passes (yellow), and low passes (pink). **(E,F)** Final pitch-passing networks constructed on a 10$\times$5 grid (50 nodes). Node size reflects total passing activity; edge thickness indicates pass frequency between regions. Red nodes highlight regions with self-loops (multiple passes within the same zone); yellow nodes indicate regions without self-loops. The network representation captures spatial organization and directional flow of ball circulation.

Our analysis integrates statistical performance indicators and network-based measures to characterize the evolution of elite football between 2020 and 2025. Figure 1 illustrates our methodological approach, showing the transformation from raw match events to the pitch-passing networks used in our analysis. The results are organized into three parts: first, we examine passing and possession dynamics; next, we explore spatial organization and gameplay patterns; and finally, we investigate pitch-passing networks to uncover shifts in spatial control and organizational strategies.

### Passing and possession dynamics

**Fig. 2:**
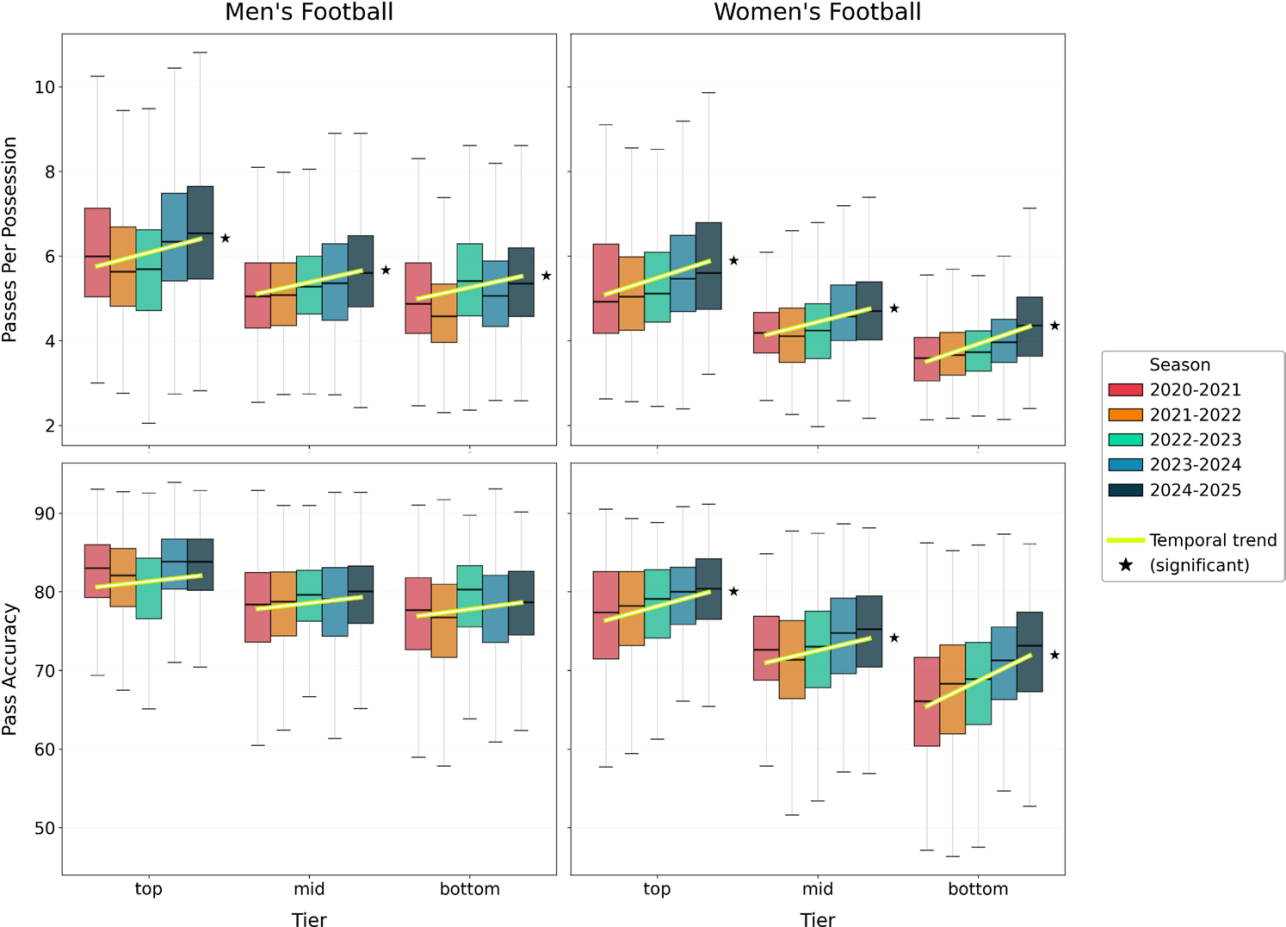
Evolution of possession intensity and passing precision. Top: passes per possession across team tiers for men’s (left) and women’s (right) football. Bottom: Overall pass accuracy across team tiers. Each panel shows distributions across five seasons (2020–2025), with temporal trends indicated by yellow lines. Asterisks ($\star$) denote statistically significant trends ($p < 0.05$ and absolute change > 5%).

Figure 2 presents the evolution of two fundamental aspects of team possession: the intensity of ball circulation (passes per possession) and passing precision (overall accuracy). Passes per possession exhibits a clear performance hierarchy across all seasons, with top-tier teams averaging approximately 6.2 passes per possession in men’s football and 5.5 in women’s football, followed by mid-tier teams (5.4 and 4.5, respectively) and bottom-tier teams (5.2 and 3.9, respectively). Notably, all tiers in both men’s and women’s competitions show significant increasing trends over the five-year period, with women’s football exhibiting particularly pronounced growth across all competitive levels (Fig. 2, top panels; Supplementary Table S4). This systematic increase suggests a league-wide shift toward more deliberate, possession-oriented play.

This pattern is also reflected in total passing volume (Supplementary Fig. S2), where top teams complete approximately 540 passes per match compared to 390–450 for bottom-tier teams. Women’s bottom-tier teams show a significant increasing trend in total passes, while other groups remain relatively stable. Additionally, passes before shot—the number of passes in sequences leading to goal attempts—exhibits consistent increases across all tiers in women’s football and modest increases in men’s football (Supplementary Figure S3), reinforcing the trend toward more elaborate attacking buildups.

Pass accuracy mirrors this performance hierarchy, with top teams achieving the highest completion rates (men: 81.9%, women: 78.2%), followed by mid-tier (men: 78.4%, women: 72.6%) and bottom teams (men: 77.4%, women: 68.7%) (Supplementary Table S5). Women’s football demonstrates stronger temporal trends, with significant increases observed in top and bottom tiers (Fig. 2, bottom panels). When examining ground passes specifically—which constitute the majority of pass attempts—accuracy ranges from 90–95% in men’s leagues to 85–90% in women’s leagues, with both showing gradual improvements over time (Supplementary Figure S4). The gender gap in passing accuracy is consistent across tiers, amounting to roughly 4 percentage points in top teams and 9 percentage points in bottom teams (Supplementary Table S6), with larger relative gaps among lower-performing teams.

**Fig. 3:**
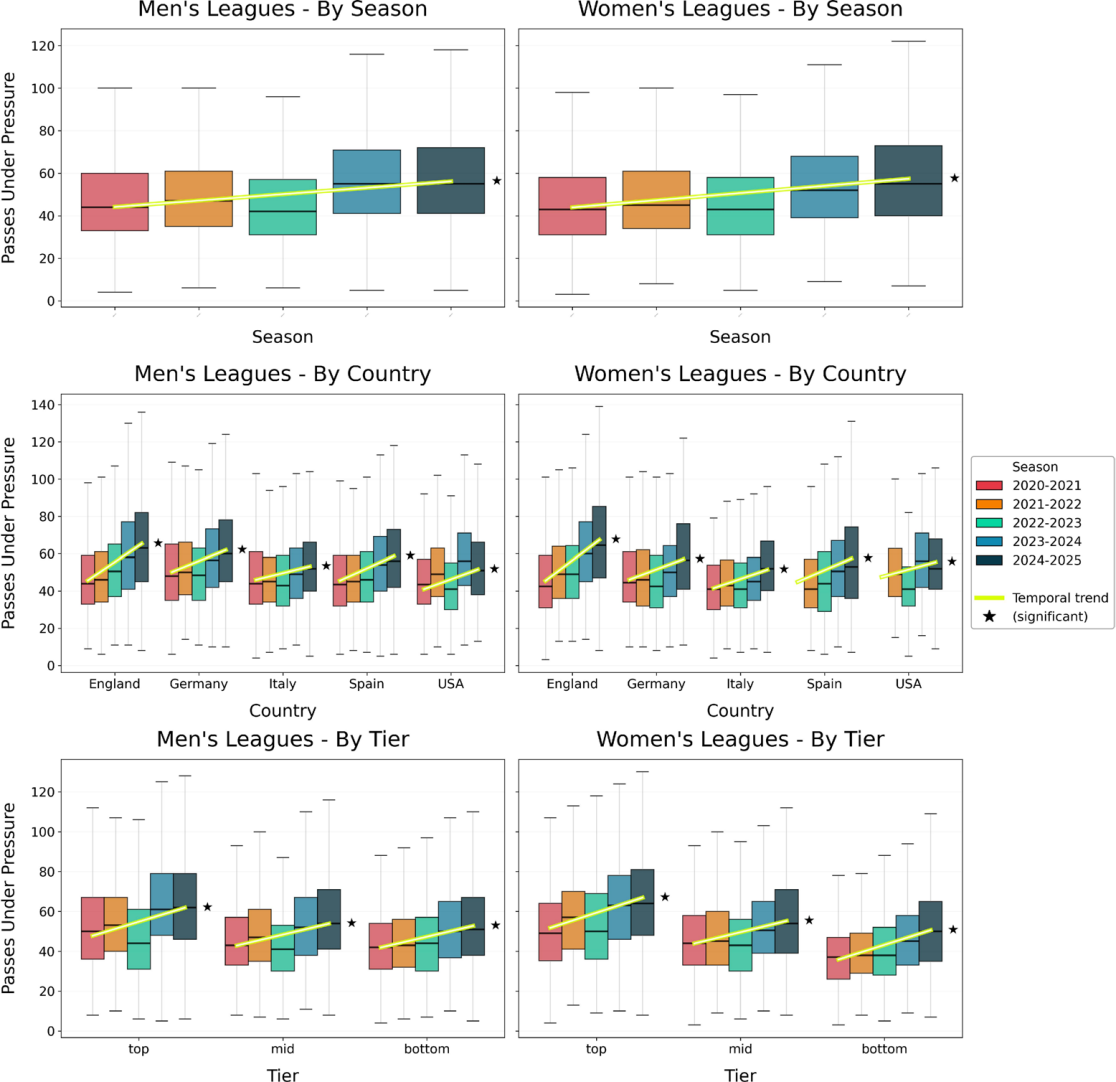
Temporal trends in passes completed under defensive pressure. Passes completed under pressure per match, shown for men’s (left) and women’s (right) football across all ten competitions. Each panel displays distributions split by season (top), by country (middle) and competitive tier (bottom). The colors indicate the season. Yellow trend lines with asterisks ($\star$) indicate significant temporal increases ($p < 0.05$ and change $> 5\%$). All the leagues show consistent upward trends across tiers, countries and genders.

The observed increase in passes completed under defensive pressure represents another measurable dimension of change in gameplay patterns. Figure 3 shows passes completed under pressure across all ten competitions, revealing substantial temporal increases in both men’s and women’s football. This trend is particularly pronounced and consistent in the English and Spanish competitions, where all tiers show significant year-over-year growth (Fig. 3, top and middle panels). The magnitude of increase is similar across genders, with both men’s and women’s competitions experiencing approximately 3–4 additional passes under pressure per match annually across all tiers (Supplementary Table S4). Notably, top women’s teams record higher absolute values than their male counterparts (60.3 vs. 55.0 passes under pressure per match; Supplementary Table S5), suggesting that the women’s elite game already operates at greater pressure intensity at the highest competitive level.

Notably, league-specific patterns emerge in the data. German and Italian women’s leagues exhibit stronger increases than their male counterparts, with the Frauen Bundesliga’s mid-tier teams showing particularly dramatic growth in the 2024–2025 season (Fig. 3, right panels). United States competitions follow a similar trend, consistent with a broad, sport-wide intensification of play under pressure.

### Spatial organization and gameplay patterns

The possession dynamics described above capture *how much* and *how accurately* teams pass, but not *where* on the pitch play unfolds. To address this, we examined three complementary aspects of spatial organization and match flow: offside positioning, directional play, and spatial pass positioning.

**Fig. 4:**
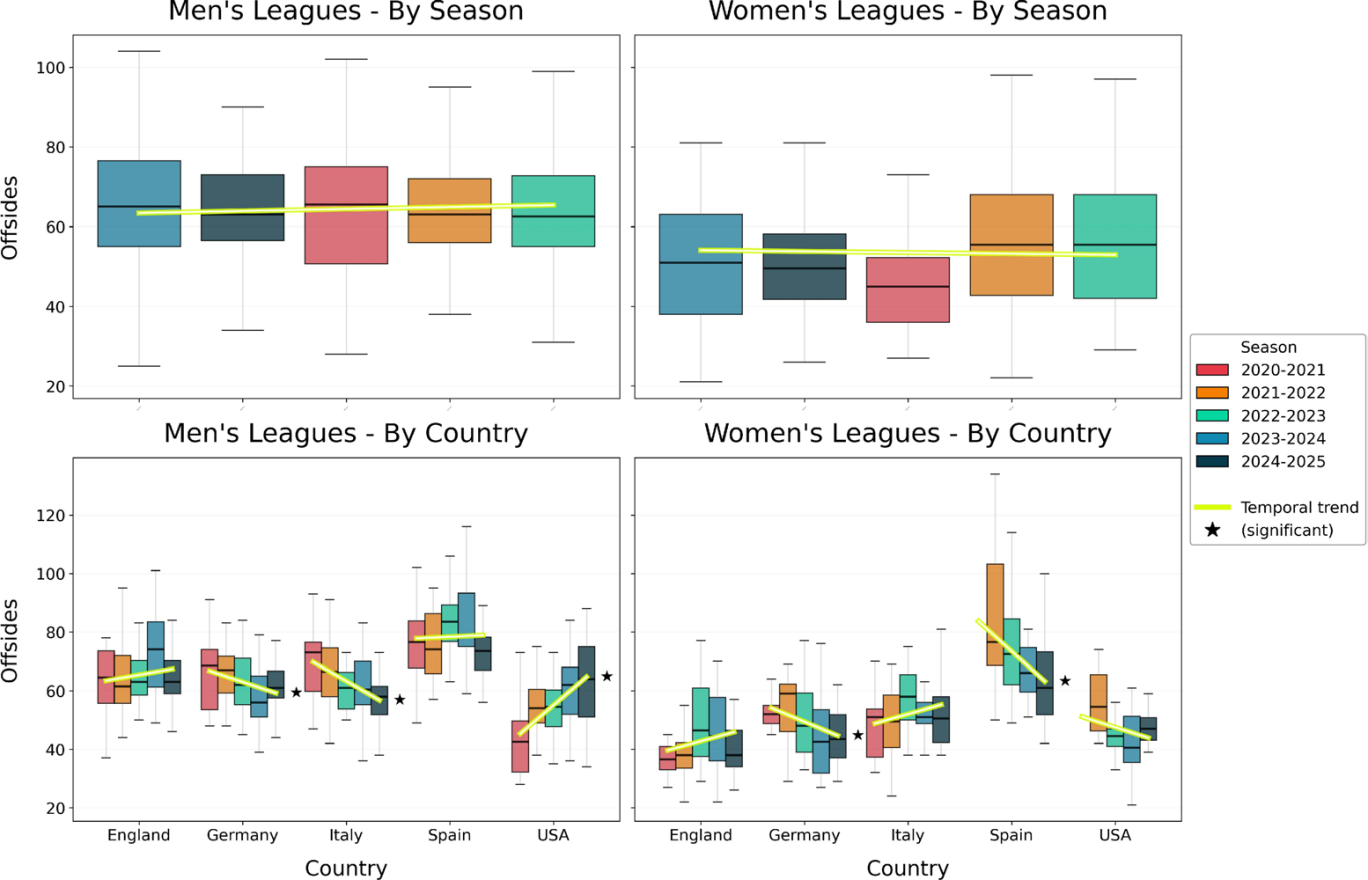
Spatial organization and gameplay dynamics. Offside infractions accumulated per team over the course of each season (i.e., summed across all matches), shown by season (top) and country (bottom). All panels compare men’s (left) and women’s (right) football across five seasons. Yellow trend lines with asterisks indicate significant changes ($p < 0.05$ and change > 5%). Note the pronounced country-level differences in offsides, with Spain exhibiting substantially higher rates than other nations.

Offside patterns exhibit clear country-level differences but minimal temporal variation (Fig. 4; Supplementary Table S7). Spanish leagues record higher offside rates than the other countries pooled in both men’s (2.06 vs 1.71, $p < 10^{-4}$) and women’s football (2.54 vs 2.10, $p < 10^{-4}$), representing an approximately 20% increase. This persistent difference is consistent with stylistic differences that have been widely discussed for Spanish football (although we do not directly measure defensive line height in this dataset).

**Fig. 5:**
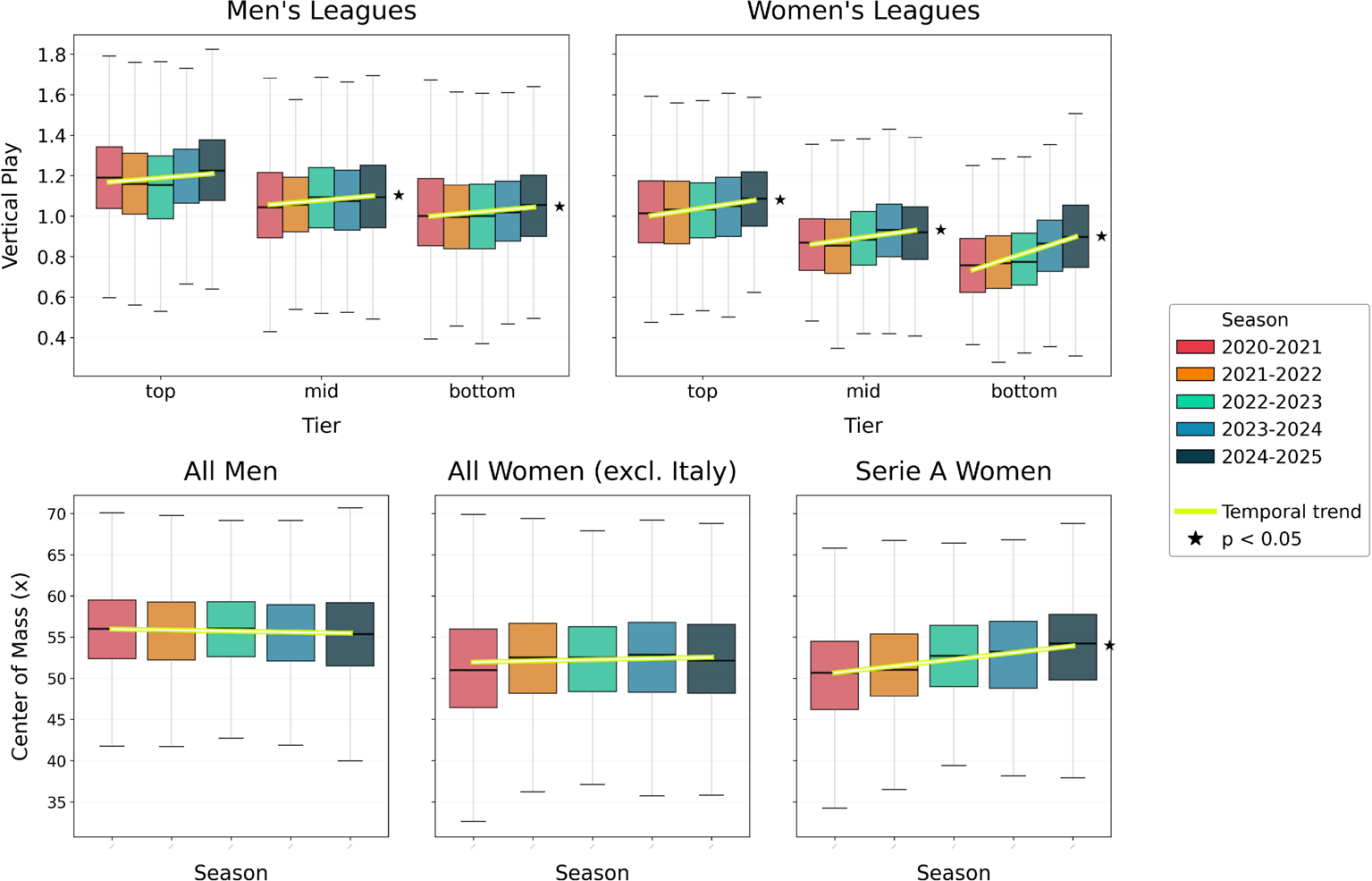
Evolution of spatial positioning: Vertical play and pass center of mass. Top row: ratio of forward-directed to lateral passes across the pitch by tier, with seasons indicated by color. Bottom row: longitudinal (x-coordinate) position of pass origins for three groups: all men’s teams (left), all women’s teams excluding Italy (center), and Serie A Women (right). A notable increase in the percentage of vertical passes is observed across seasons, competitions, and tiers. While men’s football shows declining attacking depth and other women’s leagues show minimal change, Serie A Women exhibits a strong progressive increase in forward positioning, consistent with rapid change within this competition.

Vertical play, defined as the ratio of forward-directed to lateral passes, reveals systematic differences across competitive levels (Fig. 5, top row; Supplementary Table S5). Top-tier teams exhibit the highest proportion of vertical passes in both men’s (ratio = 1.2, i.e., 20% more forward than lateral passes) and women’s (1.0) football, followed by mid-tier and bottom teams. This hierarchy is consistent with stronger teams showing a higher proportion of forward-directed passes. Temporal trends show significant increases in women’s football, particularly among top and bottom-tier teams (Supplementary Table S4), indicating an ongoing shift toward more vertical gameplay in women’s competitions. Men’s football shows more stable patterns with only modest changes over time.

Throw-in length shows modest tier-based differences and divergent temporal trends between genders, with men’s leagues exhibiting stable or increasing distances and women’s leagues displaying consistently decreasing distances (see Supplementary Fig. S6). Shot distance exhibits a consistent declining trend across most tiers in men’s football, with top and mid-tier teams showing significant decreases of approximately 0.14–0.15 m per year (Supplementary Figure S5; Supplementary Table S4). Women’s football shows similar declining patterns, though not reaching statistical significance. These trends suggest teams are working the ball closer to goal before shooting, aligning with the observed increases in passes before shot.

Spatial positioning patterns reveal distinct evolutionary trajectories across competitions. Figure 5 presents the longitudinal center of mass—the average horizontal position of successful passes—across three groups: all men’s teams, all women’s teams excluding Italy, and Serie A Women separately. Men’s football shows a modest but significant decrease in attacking depth over time (slope = -0.12 m/year, $p < 10^{-4}$), suggesting teams are initiating passes slightly deeper in their own half. Women’s football excluding Italy exhibits a small positive trend (slope = +0.15 m/year, $p = 0.03$), indicating gradual forward progression (see Supplementary Table S8).

In contrast, Italy’s Serie A Women shows a clear temporal increase (slope = +0.82 units/year, $p < 10^{-4}$), with the median longitudinal position advancing by approximately 4 units over the five-year period. This pronounced forward progression—distinct from the modest or stable trends observed in all other groups— is consistent with rapid change within this competition, coinciding with the league’s transition to full professional status in the 2022–23 season^48,49^, which eliminated the salary cap, restructured the league format, and expanded player contracts—though whether these institutional changes directly account for the observed tactical shift remains beyond the scope of the present data. More broadly, this league-specific trajectory underscores that women’s football does not follow a uniform developmental path: observable tactical differences arise through diverse, locally shaped processes, making it essential to examine individual competitions rather than treating women’s leagues as a monolithic category.

### Pitch-passing network analysis

**Fig. 6:**
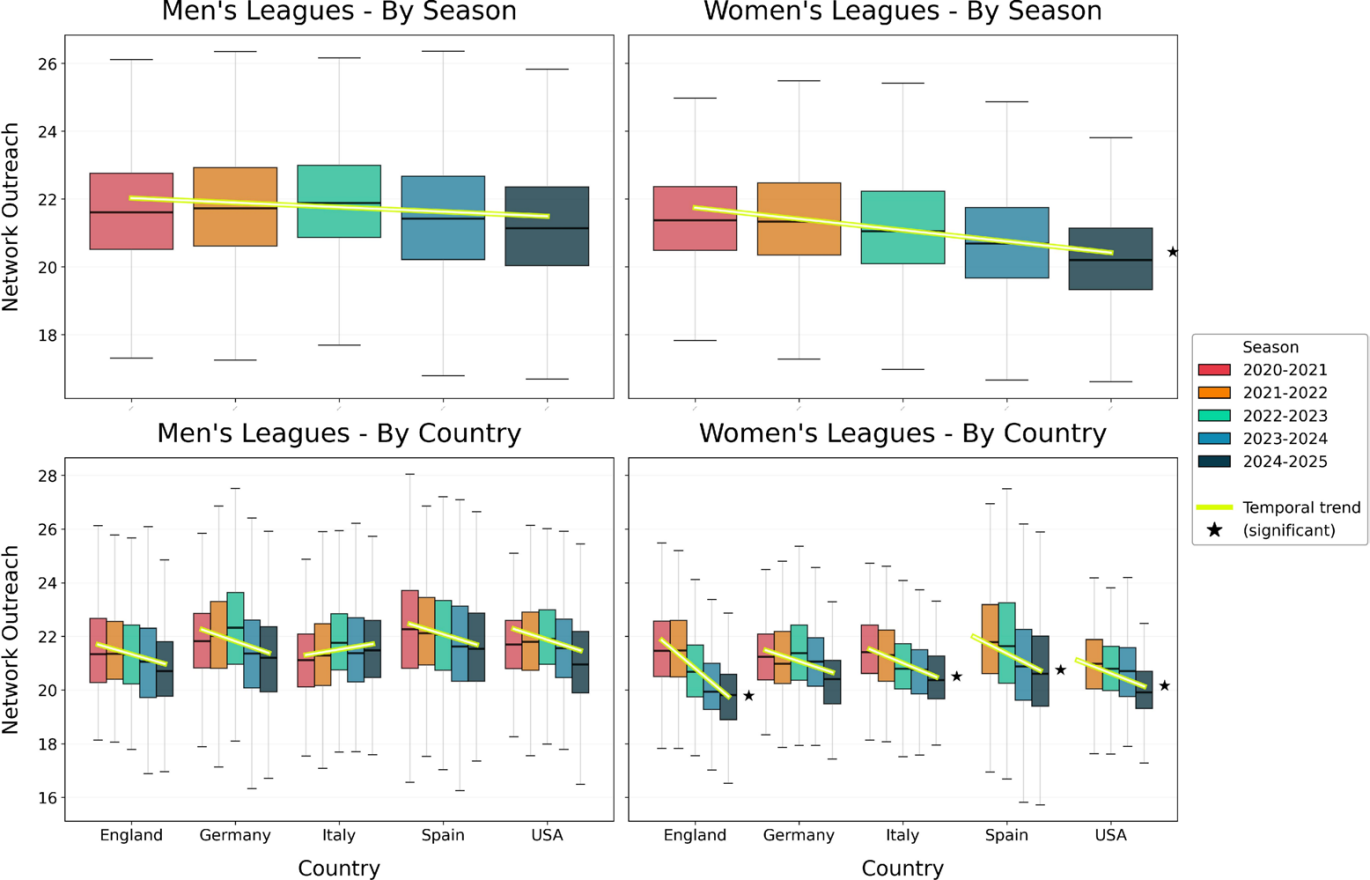
Spatial organization of ball circulation. Normalized network outreach across seasons (top panels) and countries (bottom panels) for men’s (left) and women’s (right) football. Lower outreach indicates more concentrated, shorter-range passing connections. Yellow trend lines with asterisks mark significant declining trends ($p < 0.05$ and change > 5%), most pronounced in women’s competitions. Country-level differences reveal persistent national tactical signatures, with Spain maintaining the highest spatial dispersion.

To capture the spatial organization of ball circulation, we constructed pitch-passing networks for each team and computed structural measures characterizing connectivity patterns. Figure 6 presents normalized network outreach—a measure combining passing frequency and spatial distance that quantifies how widely the ball circulates across the pitch—across seasons and countries.

Network outreach exhibits a consistent declining trend across both genders and all countries over the five-year period (Fig. 6, top panels; Supplementary Table S4). This decrease is particularly pronounced in women’s football, where all countries show significant negative trends. The temporal pattern suggests a progressive concentration of ball circulation into shorter-range passing connections rather than wide spatial distribution. This shift aligns with the observed decrease in average pass length and increase in passes per possession reported earlier, collectively suggesting a tactical evolution toward shorter, more intricate passing sequences. The decline in network outreach, as captured by Eq. 4, reflects a joint reduction in both pass distance and spatial concentration of ball circulation rather than either factor in isolation. The present event-based data do not allow us to separate these two contributions; this remains an open question for future work using tracking data.

These spatial changes are accompanied by shifts in network structural properties. Maximum eigenvalue of the adjacency matrix—reflecting connectivity strength and hierarchical organization—shows a clear tier-based hierarchy (top> mid > bottom) in both genders (Supplementary Table S5), with women’s bottom-tier teams exhibiting significant increases over time (Supplementary Table S4). Average shortest path length, quantifying circulation efficiency (Eq. 1), remains relatively stable across most groups (Supplementary Figure S7), indicating that despite more concentrated spatial distribution, teams maintain efficient connectivity across the pitch. Top teams consistently achieve the shortest path lengths (approximately 3.2–3.4), enabling rapid ball progression to any region.

Country-level patterns reveal systematic differences in spatial organization (Fig. 6, bottom panels). Spanish leagues maintain the highest network outreach in both genders, consistent with Spain’s traditional emphasis on expansive, possession-based football. In contrast, English and Italian leagues exhibit lower outreach values, reflecting more compact passing structures. These national differences remain stable across the five-year period despite the overall declining trend, indicating that while absolute outreach values decrease, relative differences between countries’ tactical styles persist.

**Fig. 7:**
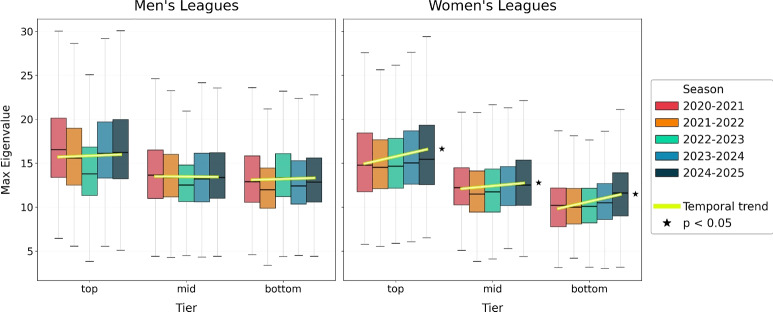
Passing network structure and hierarchical organization. Maximum eigenvalue of pitch-passing network adjacency matrix by tier for men’s (left) and women’s (right) football. Higher values indicate stronger, more centralized network structures with dominant passing routes acting as hub connections. The consistent tier gap across both genders reflects a structural signature of competitive level. Women’s bottom-tier teams exhibit a significant increasing trend, suggesting growing network cohesion at lower competitive levels.

When disaggregated by tier, an inverse relationship emerges between team performance and network outreach (Supplementary Table S5): top teams exhibit the lowest outreach (men: 21.2, women: 20.4), followed by mid-tier (men: 22.0, women: 21.2) and bottom teams (men: 22.1, women: 21.4). This inverse relationship indicates that elite performance is associated with spatially compact ball circulation rather than broad territorial coverage. Together with their higher eigenvalues and shorter path lengths (Fig. 7 and Supplementary Figure S7), these results suggest that top teams are associated with more centralized, structurally cohesive passing networks and shorter ball progression paths through compact spatial organization.

Maximum eigenvalue of the network adjacency matrix reflects overall connectivity strength and hierarchical organization (Eq. 2; Fig. 7). Top-tier teams exhibit substantially higher eigenvalues (men: 16.4, women: 15.8) than mid-tier (13.6, 12.5) and bottom-tier teams (13.1, 10.7), indicating more robust and centralized passing structures. The pronounced gap between tiers points to a structural signature of competitive level, with stronger teams organizing ball circulation around a more dominant network backbone. Women’s bottom-tier teams show a significant increasing trend, suggesting improving network cohesion at lower competitive levels.

## Discussion

Our analysis of 13,018 matches across ten elite competitions shows a systematic evolution in football gameplay between 2020 and 2025, characterized by four interconnected trends that collectively reshape how the sport is played at the highest level. First, passes completed under defensive pressure have increased substantially across all tiers and genders, consistent with a sport-wide intensification of play under pressure. This trend may reflect changes in defensive organization and pressing behavior, though the observed data do not allow us to identify the underlying mechanisms. Second, teams are working the ball progressively closer to goal before shooting, consistent with more patient, build-up oriented approaches to chance creation rather than speculative long-range attempts. Third, passes per possession continue their documented rise^29^, extending a trend observable since 2008 and indicating sustained evolution toward more elaborate buildup sequences. Fourth, network outreach has declined consistently, particularly in women’s football, reflecting more concentrated spatial ball circulation. Across all metrics, a coherent picture emerges of a broader shift in how football is played at the elite level: sustained possession through shorter, more intricate passing sequences appears increasingly prevalent, alongside intensified pressing when out of possession—trends observable across the coaching philosophies documented in contemporary football literature^50^.

The observed improvements in passing accuracy—particularly in ground passes, which constitute the majority of attempts—likely reflect multiple interacting factors, including tactical preferences (e.g., shorter passing sequences), technical training, and coaching emphasis. Because we do not directly measure pitch conditions, training environments, or rule and officiating changes over time, we avoid attributing these trends to any single mechanism. That said, pitch quality improvements over the study period likely contribute: modern elite stadiums employ sophisticated drainage systems, hybrid grass-synthetic surfaces, and intensive maintenance regimes that maintain consistent playing surfaces, facilitating the execution of technical skills, which may partly account for the observed improvements in passing precision. The gender gap in passing accuracy amounts to roughly 4 percentage points in top-tier teams and 9 percentage points in bottom-tier teams (Supplementary Table S6), which may reflect not only differences in technical development pathways but also potential disparities in training facility quality and pitch maintenance standards between men’s and women’s competitions, though the latter is rapidly improving as women’s football professionalization accelerates.

Women’s football exhibits particularly pronounced rates of change across multiple metrics, consistent with a competition undergoing rapid professionalization and tactical development^3^. The magnitude of temporal trends in women’s competitions often exceeds those observed in men’s football, suggesting that the women’s game is undergoing particularly rapid change as leagues mature, coaching methodologies develop, and player development pathways expand. Critically, our analysis reveals substantial heterogeneity in these evolutionary trajectories. Italy’s Serie A Women demonstrates a pronounced forward progression in pass positioning (Fig. 5; Supplementary Table S8)—advancing approximately four meters upfield over five seasons—distinct from patterns observed in other women’s competitions. This league-specific modernization underscores that women’s football does not follow a uniform developmental path but rather exhibits distinct trajectories shaped by local investment patterns, coaching philosophies, and institutional structures. Beyond its descriptive value, this heterogeneity carries methodological implications: aggregating women’s leagues into a single category risks masking divergent evolutionary dynamics and may lead to misleading cross-gender comparisons. We acknowledge that several of our own analyses aggregate all women’s competitions for comparability with men’s football; these aggregated results should be read as identifying broad dataset-level patterns rather than as claims about any individual league. As women’s football continues to professionalize at different rates across countries, future research and policy decisions—including resource allocation, coaching certification standards, and league governance—would benefit from frameworks that treat individual competitions as distinct systems rather than instances of a common template. The league’s transition to full professional status in the 2022–23 season^48,49^ provides a natural quasi-experimental setting for future work examining whether institutional restructuring is associated with measurable changes in playing style.

Network analysis reveals that elite performance is associated with qualitatively different spatial organization rather than mere quantitative superiority. Top teams construct more centralized, efficient passing networks—evidenced by higher eigenvalues and shorter path lengths—yet paradoxically exhibit lower network outreach than bottom-tier teams. This inverse relationship suggests that higher competitive performance is associated with more compact, concentrated ball circulation—consistent with deliberate short-passing buildup strategies—rather than wide spatial distribution, which may instead reflect less controlled, longer-range ball movement in lower-performing teams. The declining outreach observed across all competitions indicates a sport-wide shift: teams increasingly show patterns of controlled possession in concentrated areas over expansive territorial coverage.

Country-level differences reveal that while tactical evolution affects all competitions, national styles remain distinguishable throughout. Spain’s persistently higher offside rates and network outreach across both genders are consistent with widely documented stylistic tendencies toward high defensive lines and expansive possession play^32^. These stable national signatures, maintained despite overall league-wide trends, suggest that football’s tactical evolution operates within culturally constrained boundaries—leagues modernize and intensify, yet distinctive national identities persist.

Several limitations warrant acknowledgment and possibly further research. Our five-season analytical window, while capturing recent evolution, may not reveal longer-cycle tactical shifts that characterize multi-decade developments in established men’s leagues. The proprietary nature of event data, though necessary for comprehensive analysis, may limit reproducibility. We discuss the potential influence of *effective playing time*—the total time the ball is actively in play during a match—on count-based metrics in the Supplementary Information, where we show that the main findings are robust to normalization by passing volume as a proxy for ball-in-play duration^51–53^. Future research should extend the temporal scope to contextualize recent changes within longer historical trajectories, investigate team-specific tactical signatures that may be obscured in league-level aggregation using advanced network motif analysis^16,17^, and explore whether observed trends predict competitive success or represent stylistic preferences independent of outcomes^18^. The rapid transformation of women’s football—marked by accelerating professionalization, increasing investment, and evolving tactical sophistication—merits dedicated longitudinal study to document this historic developmental phase. Additionally, institutional structures such as ownership models^54^ and wearable technology adoption^28^ may influence tactical evolution in ways not captured by our analysis. Our analysis is descriptive in nature and aims to characterize sport-wide and league-level evolutionary trends rather than to predict match outcomes or isolate individual team styles. Match-level metrics are aggregated across all game states, regardless of scoreline, game phase, or opposition strength; while this approach is appropriate for identifying broad temporal trends at the league level, it may not control for situational confounders that may influence passing behavior in individual matches. While the primary aim of this work is descriptive, the league-wide trends documented here may offer a secondary point of reference for practitioners seeking to characterize and contextualize the evolving tactical landscape of elite football. We leave the translation of these structural patterns into actionable recommendations to future applied work.

Overall, our results provide quantitative evidence of recent shifts in possession, pressure, and spatial passing structure across multiple elite competitions. The consistency of several trends across countries and tiers suggests that recent tactical changes are not confined to a single league, while persistent cross-country differences indicate that stylistic diversity remains.

## Methods

### StatsBomb events data

The data analyzed in this work, provided under license from Hudl StatsBomb^42^, comprise 13,018 matches played between 2020 and 2025 across ten top-tier competitions in five countries (Supplementary Table S1). The dataset consists of event data—timestamped records of all on-pitch actions including their Euclidean coordinates and outcomes—covering passes, shots, fouls, substitutions, and other match events. Our analysis focuses primarily on passing and shooting actions, as these capture the fundamental dynamics of ball movement and goal-scoring opportunities.

We classified teams into three performance tiers (top, mid, bottom) based on percentile ranks of final league standings within each season and competition, dividing teams into approximately equal thirds. This classification enables comparative analysis of tactical evolution across different competitive levels while accounting for varying league sizes. This tier classification is defined within each competition and season separately, and comparisons across leagues should therefore be interpreted with caution: tier labels are intended to capture relative competitive standing within each competition rather than absolute tactical or technical level. Similarly, analyses aggregating all women’s competitions are intended to identify broad patterns across the dataset; where between-league heterogeneity is salient—as in the case of Serie A Women (Fig. 5)—individual competitions are examined separately. We caution against interpreting aggregated women’s results as representative of any single league.

### Statistical analysis

We computed a comprehensive set of team-level performance metrics for each match, encompassing passing dynamics, shooting behavior, spatial positioning, and game flow characteristics. From this initial set, we selected metrics that exhibited meaningful variation, were non-redundant with one another, and provided insight into the tactical and strategic evolution of football. A complete list of all computed statistical features is provided in Supplementary Table S2.

Additionally, we derived several spatial and tactical features: (i) *pass center of mass* (mean *x*-coordinate of completed pass origins), capturing average attacking depth; (ii) *vertical play*, defined as the ratio of forward-directed to lateral passes, where a pass is classified as forward if its displacement along the pitch axis (x-component) exceeds its lateral displacement (y-component), and as lateral otherwise; and (iii) *offsides* (number of offside infractions per team per season).

Our statistical analysis addressed three complementary questions. First, to assess temporal evolution within tiers, we performed linear regression for each combination of gender and tier (top, mid, bottom), using season year as the independent variable and the metric of interest as the dependent variable. Regression was performed on all individual team-season observations rather than aggregated means, preserving the full distribution of the data. This approach quantifies whether performance indicators changed systematically over the five-year period (2020-2025) and whether the rate of change differed across competitive levels. In visualizations, temporal trends are marked as statistically significant (indicated by $\star$) only when both $p < 0.05$ and the absolute percent change over the five-year period exceeded 5%.

Second, to evaluate differences among tiers, we conducted one-way ANOVA separately for men’s and women’s competitions, testing whether mean metric values differed significantly across the three tiers within each gender. Third, to compare gender differences by tier, we performed independent-samples t-tests for each tier, comparing men’s and women’s teams at the same competitive level. For each comparison, we report the mean values for both groups, the absolute difference, and the percent difference calculated as $\Delta \% = 100 \times (M - W) / W$, where positive values indicate higher performance in men’s football.

All statistical tests were two-tailed with significance threshold set at $p < 0.05$. Results are summarized in Tables S4, S5, and S6 with detailed outputs provided in the Supplementary Information. Given the number of tests conducted across metrics, tiers, and genders, we assessed robustness to multiple comparisons by applying Benjamini–Hochberg correction within each metric family. Corrected *p*-values are reported in Supplementary Table S9.

### Pitch network

#### Network construction

To complement statistical performance indicators, we constructed pitch-passing networks for each team in every match. This network-based representation has been successfully applied in previous studies to identify playing styles, quantify spatial organization, and reveal tactical patterns not readily apparent from conventional statistics^43–47^.

The pitch was discretized into a regular grid of spatial zones using StatsBomb’s standardized $120 \times 80$ coordinate system. Following established methodologies^45^, we employed a $10 \times 5$ grid structure, yielding 50 spatial regions (Supplementary Figure S1). This configuration aligns with prior work demonstrating that approximately 49 regions represent an optimal balance between spatial resolution and statistical reliability for pitch network analysis^45^. Each region serves as a node in the network, while directed, weighted edges connect regions between which successful passes occur. Edge weights correspond to the number of passes exchanged between regions throughout the match, capturing both the frequency and directionality of ball movement across the pitch.

To facilitate comparative analysis across matches with different passing volumes, we normalized edge weights by the total network strength (sum of all edge weights), yielding a representation in which link weights sum to 100. This normalization ensures that network metrics reflect structural properties of ball circulation rather than overall passing volume. Figure 1 illustrates this construction process for a representative match between Arsenal and Liverpool.

#### Network analysis

We computed network-level metrics that characterize the structural properties of ball circulation patterns. All metrics are computed on directed, weighted graphs, preserving the directionality of passes between pitch regions. *Average shortest path length* quantifies the average minimum number of passes required to connect any two regions, with lower values indicating more direct ball progression. Average shortest path length is computed on the largest strongly connected component of the directed network using Dijkstra’s algorithm, ensuring that path lengths are well-defined for all node pairs. Formally:

1 $$\begin{aligned} L = \frac{1}{|V|(|V|-1)} \sum _{i \ne j} d(i, j) \end{aligned}$$

where *d*(*i*, *j*) is the shortest path length from node *i* to node *j* computed via Dijkstra’s algorithm with the inverse of edge weights as distances, so that frequently used connections are treated as shorter paths. The metric is computed on the largest strongly connected component of the directed network, ensuring that *d*(*i*, *j*) is well-defined for all node pairs. The *maximum eigenvalue* of the adjacency matrix reflects the network’s overall connectivity and hierarchical structure, with higher values suggesting more centralized passing patterns. Formally, it corresponds to the spectral radius of the weighted adjacency matrix *A*:

2 $$\begin{aligned} \lambda _{\max } = \max \left\{ |\lambda | : \lambda \in \sigma (A) \right\} \end{aligned}$$

where $\sigma (A)$ denotes the spectrum of *A*. In spatially embedded weighted networks—such as pitch-passing networks—where links carry both a weight and a physical distance, it is possible to quantify how connection strength is distributed across space. The *network outreach* of a node *i* is defined as

3 $$\begin{aligned} O_i = \sum _{j \in \mathcal {N}(i)} w_{ij} \, d_{ij}, \end{aligned}$$

where $w_{ij}$ denotes the weight of the connection between nodes *i* and *j*, $d_{ij}$ is the Euclidean distance between them, and $\mathcal {N}(i)$ represents the set of neighbors of node *i*. The mean outreach of the network is then $O = \frac{1}{N} \sum _i O_i$. Originally introduced in the context of brain spatial networks^55^, this quantity captures the joint contribution of connection strength and spatial embedding, measuring how weighted connectivity is allocated to long- versus short-range links. High outreach indicates that a significant fraction of total connection weight is carried by long-range links; conversely, low outreach implies that connectivity is dominated by short-range interactions, yielding more locally clustered, spatially compact network architectures. In the context of pitch-passing networks, we adapt this measure to directed networks as follows. *Outreach* measures the spatially weighted dispersion of passes, calculated as the sum of pass counts multiplied by the Euclidean distance between connected regions, normalized by total network strength; higher outreach indicates longer-range ball circulation across the pitch. Formally:

4 $$\begin{aligned} \text {Outreach} = \frac{1}{|V|} \sum _{i \in V} \sum _{j \in \mathcal {N}^+(i)} w_{ij} \cdot \left\| {\bf c}_i - {\bf c}_j \right\| _2 \end{aligned}$$

where $w_{ij}$ is the normalized weight of the directed edge from node *i* to node *j*, ${\bf c}_i$ and ${\bf c}_j$ are the centroids of pitch zones *i* and *j*, and $\mathcal {N}^+(i)$ denotes the out-neighbours of node *i*. Because $w_{ij}$ encodes both passing frequency and spatial reach jointly, a decline in outreach reflects a reduction in the spatially weighted distribution of passes—capturing simultaneously shorter pass distances and a more concentrated spatial distribution of ball circulation, rather than either factor in isolation.

These network metrics were aggregated at the team-season level and analyzed across tiers, genders, and countries following the same statistical framework described above. Complete descriptions of all network measures are provided in Supplementary Table S3.

## Supplementary Information

Supplementary Information.

## Data Availability

Although the data used in this study are not publicly available (provided under license from Hudl StatsBomb), an equivalent surrogate dataset (same provider and same format) can be found in the StatsBomb open-data repository (https://github.com/statsbomb/open-data). The code used for analysis and figure generation is available at https://github.com/maddaleona/harder_shorter_sharper_forward/.

## References

[1] Williams, J. *A Game for Rough Girls? A History of Women’s Football in Britain* (Routledge (2003).

[2] Lopez, S. *Women on the Ball: A Guide to Women’s Football* (Scarlet Press, 1997).

[3] FIFA. Women’s Football Strategy. https://inside.fifa.com/womens-football/strategy (2023).

[4] Bridge, T., Haskel, J. & Dow, F. Annual review of football finance: Women’s super league. https://www.deloitte.com/uk/en/services/consulting/research/annual-review-of-football-finance-womens-super-league.html (2025).

[5] Duch, J., Waitzman, J. S. & Amaral, L. A. N. Quantifying the performance of individual players in a team activity. *PLOS ONE* **5**, e10937. 10.1371/journal.pone.0010937 (2010). 20585387 10.1371/journal.pone.0010937 PMC2886831

[6] Fewell, J. H., Armbruster, D., Ingraham, J., Petersen, A. & Waters, J. S. Basketball teams as strategic networks. *PLOS ONE* **7**, e47445. 10.1371/journal.pone.0047445 (2012). 23139744 10.1371/journal.pone.0047445 PMC3490980

[7] Tennant, A. G., Smith, C. M. L. & Chen, J. E. C. Who was the greatest of all-time? A historical analysis by a complex network of professional boxing. *J. Complex Netw.* **8**, cnaa009. 10.1093/comnet/cnaa009 (2020).

[8] Gómez, M. -Á., Rivas, F., Leicht, A. S. & Buldú, J. M. Using network science to unveil badminton performance patterns. *Chaos Solit. Fract.* **135**, 109834. 10.1016/j.chaos.2020.109834 (2020).

[9] Cintia, P., Coscia, M. & Pappalardo, L. The Haka network: Evaluating rugby team performance with dynamic graph analysis. In 2016 IEEE/ACM International Conference on Advances in Social Networks Analysis and Mining (ASONAM). 1095–1102 (IEEE, 2016).

[10] Passos, P. et al. Networks as a novel tool for studying team ball sports as complex social systems. *J. Sci. Med. Sport* **14**, 170–176. 10.1016/j.jsams.2010.10.459 (2011). 21145787 10.1016/j.jsams.2010.10.459

[11] Ribeiro, J., Silva, P., Duarte, R., Davids, K. & Garganta, J. Team sports performance analysed through the lens of social network theory: Implications for research and practice. *Sports Med.* **47**, 1689–1696. 10.1007/s40279-017-0695-1 (2017). 28197801 10.1007/s40279-017-0695-1

[12] Buldú, J. M. et al. Using network science to analyse football passing networks: Dynamics, space, time, and the multilayer nature of the game. *Front. Psychol.* **9**, 1900. 10.3389/fpsyg.2018.01900 (2018). 30349500 10.3389/fpsyg.2018.01900 PMC6186964

[13] Ramos, J., Lopes, R. J. & Araújo, D. What’s next in complex networks? Capturing the concept of attacking play in invasive team sports. *Sports Med.* **48**, 17–28. 10.1007/s40279-017-0786-z (2018). 10.1007/s40279-017-0786-z 28918464

[14] Pan, P., Lago Peñas, C., Wang, Q. & Liu, T. Evolution of passing network in the soccer world cups 2010–2022. *Sci. Med. Football* **9**, 349–360. 10.1080/24733938.2024.2386359 (2025). 10.1080/24733938.2024.2386359 39105667

[15] Peña, J. L. & Touchette, H. A network theory analysis of football strategies. arXiv 10.48550/arXiv.1206.6904 (2012).

[16] Li, M.-X., Xu, L.-G. & Zhou, W.-X. Motif analysis and passing behavior in football passing networks. *Chaos Solit. Fract.* **190**, 115750. 10.1016/j.chaos.2024.115750 (2025).

[17] Aguirre, J., Papo, D. & Buldú, J. M. Successful strategies for competing networks. *Nat. Phys.* **9**, 230–234. 10.1038/nphys2556 (2013).

[18] Basini, F., Tsouli, V., Ntzoufras, I. & Friel, N. Assessing competitive balance in the English premier league for over forty seasons using a stochastic block model. *J. R. Stat. Soc. Ser. A Stat. Soc.* **186**, 530–556. 10.1093/jrsssa/qnad007 (2023).

[19] Pappalardo, L. & Cintia, P. Quantifying the relation between performance and success in soccer. *Adv. Complex Syst.* **21**, 1750014. 10.1142/S021952591750014X (2018).

[20] Pappalardo, L. et al. Playerank: Data-driven performance evaluation and player ranking in soccer via a machine learning approach. *ACM Trans. Intell. Syst. Technol.* **10**, 59:1–59:27. 10.1145/3343172 (2019).

[21] Pappalardo, L. et al. A public data set of spatio-temporal match events in soccer competitions. *Sci. Data* **6**, 236. 10.1038/s41597-019-0247-7 (2019). 31659162 10.1038/s41597-019-0247-7 PMC6817871

[22] Buldú, J. M. et al. Football tracking networks: Beyond event-based connectivity. arXiv:2011.06014 (2020).

[23] Buldú, J. M., Busquets, J., Echegoyen, I. & Seirullo, F. Defining a historic football team: Using network science to analyze Guardiola’s FC Barcelona. *Sci. Rep.* **9**, 13602. 10.1038/s41598-019-49969-2 (2019). 10.1038/s41598-019-49969-2 PMC6753100 31537882

[24] Spearman, W., Basye, A., Dick, G., Hotovy, R. & Pop, P. Physics-based modeling of pass probabilities in soccer. In *Proceedings of the 11th MIT Sloan Sports Analytics Conference* (2017).

[25] Fernández, J. & Bornn, L. Wide open spaces: A statistical technique for measuring space creation in professional soccer. In *Proceedings of the Sloan Sports Analytics Conference* (2018).

[26] Rico-González, M., Pino-Ortega, J., Méndez, A., Clemente, F. M. & Baca, A. Machine learning application in soccer: A systematic review. *Biol. Sport* **40**, 249–263. 10.5114/biolsport.2023.112970 (2023). 36636183 10.5114/biolsport.2023.112970 PMC9806754

[27] Biermann, H. et al. Synchronization of passes in event and spatiotemporal soccer data. *Sci. Rep.* **13**, 15878. 10.1038/s41598-023-39616-2 (2023). 37741829 10.1038/s41598-023-39616-2 PMC10518005

[28] Li, R. T. et al. Wearable performance devices in sports medicine. *Sports Health* **8**, 74–78. 10.1177/1941738115616917 (2016). 26733594 10.1177/1941738115616917 PMC4702159

[29] González-Rodenas, J. et al. Evolution of tactics in professional soccer: An analysis of team formations from 2012 to 2021 in the Spanish Laliga. *J. Hum. Kinet.* **88**(207–216), 2023. 10.5114/jhk/167468 (2012). 10.5114/jhk/167468 PMC10407311 37559775

[30] González-Rodenas, J., Moreno-Pérez, V., López-Del Campo, R., Resta, R. & Del Coso, J. Technical and tactical evolution of the offensive team sequences in Laliga between 2008 and 2021. Is Spanish football now a more associative game? *Biol. Sport* **40**. 10.5114/biolsport.2024.131818 (2024). 10.5114/biolsport.2024.131818 PMC10955746 38524831

[31] Bauer, P., Anzer, G. & Shaw, L. Putting team formations in association football into context. *J. Sports Anal.* **9**, 39–59. 10.3233/JSA-220620 (2023).

[32] Wilson, J. *Inverting the Pyramid: The History of Football Tactics* (Orion, 2008).

[33] Goldblatt, D. *The Game of Our Lives: The Meaning and Making of English Football* (Penguin, 2014).

[34] Ayana, G. et al. Temporal dynamics of goal scoring in soccer. arXiv:2501.18606 (2025).

[35] Power, L. *The Changing Landscape of Ownership in the Premier League* (Premier League Now, 2025).

[36] Noble, J. European football club takeovers halve in a year. *Financ. Times* (2025).

[37] Bauers, S. B. & Hovemann, G. The regulation of investors’ influence in German professional football. *German J. Exerc. Sport Res.* **49**, 463–471. 10.1007/s12662-019-00595-0 (2019).

[38] Timms, A. MLS year 30: A league at a philosophical crossroads as world cups loom. *Guardian* (2025).

[39] Rigby, S., Wills, A., Maturi, T. & O’Malley, C. Keeping possession: Ownership trends in English premier league football. https://www.nortonrosefulbright.com/en/knowledge/publications/d6fe3b17/ownership-structure-of-womens-football-in-the-uk (2023).

[40] Pappalardo, L., Rossi, A., Natilli, M. & Cintia, P. Explaining the difference between men’s and women’s football. *PLOS ONE* **16**, e0255407. 10.1371/journal.pone.0255407 (2021). 34347829 10.1371/journal.pone.0255407 PMC8336886

[41] Molango, M. Club world cup creates unsustainable never-ending calendar. *Times* (2025).

[42] StatsBomb Services Ltd. StatsBomb Football Data (Private Dataset). Access provided under institutional license. https://statsbomb.com (2025).

[43] Zhou, W., Yu, G., You, S. & Wang, Z. An improved passing network for evaluating football team performance. *Appl. Sci.* **13**, 845. 10.3390/app13020845 (2023).

[44] Herrera-Diestra, J. L. et al. Pitch networks reveal organizational and spatial patterns of Guardiola’s FC Barcelona. *Chaos Solit. Fract.* **138**, 109934. 10.1016/j.chaos.2020.109934 (2020).

[45] Garrido, D. et al. Consistency and identifiability of football teams: A network science perspective. *Sci. Rep.* **10**, 19735. 10.1038/s41598-020-76835-3 (2020). 33184412 10.1038/s41598-020-76835-3 PMC7661721

[46] Novillo, Á. et al. A multilayer network framework for soccer analysis. *Chaos Solit. Fract.* **178**, 114355. 10.1016/j.chaos.2023.114355 (2024).

[47] Huang, K., Gong, B., Xu, K. & Zhou, C. Revealing pitch-passing network differences between uefa euro 2024 quarterfinalists and non-quarterfinalists. In *Proceedings of the 2024 International Conference on Sports Technology and Performance Analysis*. 204–212. 10.1145/3723936.3723967 (2024).

[48] FIGC. Giornata Storica: Il Calcio Femminile Passa Oggi Al Professionismo (2022).

[49] *What the Professionalization of Serie A Femminile Means for Women’s Soccer in Italy Equalizer Soccer* (2023).

[50] Violán, M. À. *Pep Guardiola: The Philosophy That Changed the Game* (Meyer & Meyer, 2014).

[51] Spyrou, K. et al. Interpreting match performance in elite futsal: Considerations for normalizing variables using effective time. *Front. Sports Active Living* **5**, 1256424. 10.3389/fspor.2023.1256424 (2023). 10.3389/fspor.2023.1256424 PMC10507323 37731478

[52] Altmann, S., Forcher, L., Woll, A. & Härtel, S. Effective playing time affects physical match performance in soccer: An analysis according to playing position. *Biol. Sport* **40**, 967–973. 10.5114/biolsport.2023.123320 (2023). 37867750 10.5114/biolsport.2023.123320 PMC10588592

[53] Tojo, Ó., Spyrou, K., Teixeira, J., Pereira, P. & Brito, J. Effective playing time affects technical-tactical and physical parameters in football. *Front. Sports Active Living* **5**, 1229595. 10.3389/fspor.2023.1229595 (2023). 10.3389/fspor.2023.1229595 PMC10442814 37614412

[54] Budzinski, O. & Kunz-Kaltenhäuser, P. Promoting or restricting competition? The 50plus1-rule in German football. In *Ilmenau Economics Discussion Papers*. Vol. 26. 10.2139/ssrn.3623779 (2020).

[55] Buldú, J. M. et al. Reorganization of functional networks in mild cognitive impairment. *PLoS ONE* **6**, e19584. 10.1371/journal.pone.0019584 (2011). 21625430 10.1371/journal.pone.0019584 PMC3100302

