# Supplementary material for: Evolution of spatial structure, passing network patterns, and gameplay intensity in elite women’s and men’s football (2020–2025)
Source: Sci Rep. 2026 May 14;16:22007. doi: 10.1038/s41598-026-52701-6 (PMC13365384; doi:10.1038/s41598-026-52701-6)
Supplement: Supplementary file 1 — Supplementary Information. [file 41598_2026_52701_MOESM1_ESM.pdf]

Supplementary Information for: Evolution of spatial structure, passing network patterns, and gameplay intensity in elite women's and men's football (2020-2025)

Rebecca Carstens[1], Raj Deshpande[2], Pau Esteve[3], Nicolò Fidelibus[4], Sara Linde Neven[5], Ramona Ottow[6], Lokamruth K. R.[7], Paula Rodríguez-Sánchez[8], Luca Santagata[9,10], Javier M. Buldú[8], Brennan Klein[10,11,12], Maddalena Torricelli[10,*]

Dataset composition

Table 1 provides a detailed breakdown of the 13,018 matches analyzed in this study, organized by country and gender. The dataset spans five seasons (2020-2025) and encompasses both established leagues with stable team numbers (European competitions) and expanding leagues (MLS and NWSL in the United States).

Pitch network construction

The construction of pitch-passing networks follows a spatial discretization approach in which the football pitch is divided into regular zones that serve as network nodes. Figure 1 illustrates the 10×5 grid structure employed in this study, yielding 50 spatial regions across the standardized 120m × 80m pitch dimensions. Nodes are numbered sequentially from left to right (attacking direction), with rows representing different lateral positions on the pitch. Each successful pass between regions creates a directed edge in the network, with edge weight corresponding to the number of passes exchanged between those regions throughout the match.

Performance metrics

We computed a comprehensive set of performance indicators from match event data, encompassing both conventional match statistics and network-derived measures of spatial ball circulation. Tables 2 and 3 provide complete specifications for statistical and network-based measures, respectively, distinguishing between metrics reported in the main analysis and those computed for exploratory purposes.

Statistical metrics

In Table 2, we list the statistical measures analyzed in this work.

Country	Competition	Men	Women	Total
Germany	Bundesliga / Frauen Bundesliga	1,529	652	2,181
Spain	La Liga / Liga F	1,900	927	2,827
England	Premier League / FA WSL	1,899	659	2,558
Italy	Serie A / Serie A Women	1,900	620	2,520
USA	MLS / NWSL	2,276	656	2,932
**Total**		**9,504**	**3,514**	**13,018**

Table 1. Supplementary Table S1. Number of matches analyzed by country and gender (2020-2025). MLS expanded from 26 to 30 teams during this period; NWSL expanded from 10 to 14 teams. Liga F coverage began in the 2021-22 season.

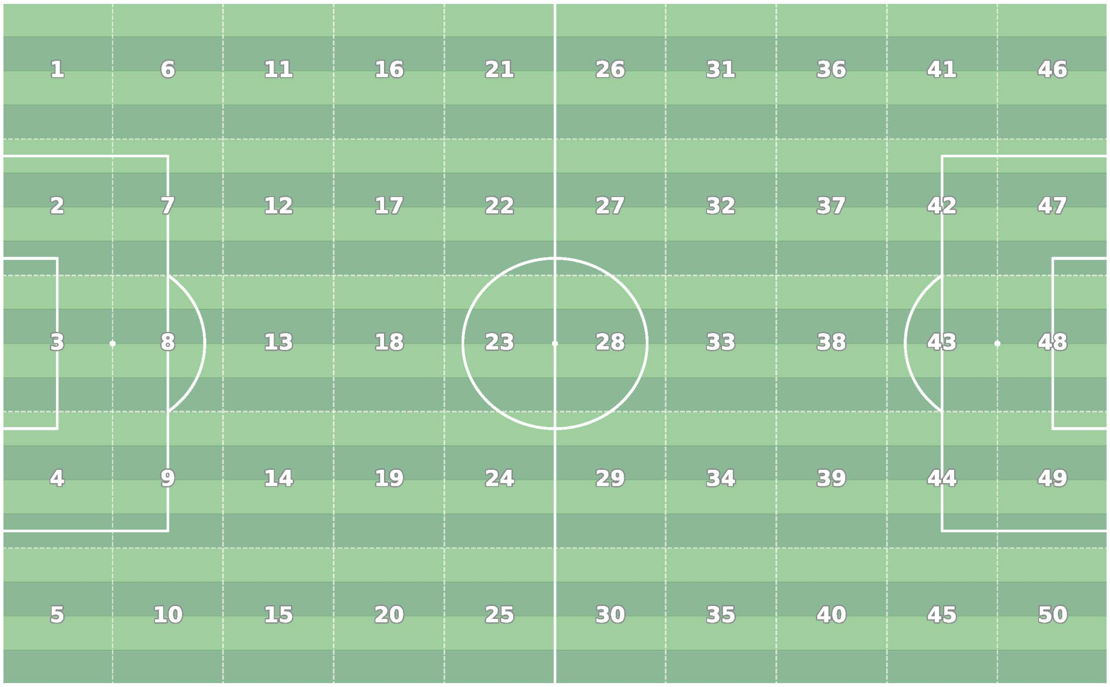

Figure 1. Supplementary Figure S1. Spatial discretization of the football pitch for network construction. The pitch is divided into a 10×5 regular grid creating 50 zones numbered sequentially from the defensive third (left, nodes 1-10) to the attacking third (right, nodes 41-50). Each zone serves as a node in the pitch-passing network, with directed edges connecting zones between which successful passes occur. Edge weights correspond to the number of passes exchanged between regions throughout the match.

Metric	Description	Unit
Reported in article		
Number of passes	Total number of passes attempted by the team during the match.	count
Passes per possession (PPP)	Average number of completed passes during each team possession phase; indicates possession style (buildup vs. direct play).	passes
Pass accuracy	Percentage of attempted passes that were successfully completed.	%
Ground pass accuracy	Percentage of ground-level passes that were successfully completed.	%
Passes before shot (PBS)	Average number of passes completed by the team in the sequence leading to each shot attempt.	passes
Passes under pressure	Number of completed passes made while the passer was under defensive pressure.	count
Shot distance	Average Euclidean distance from shot initiation location to the center of the goal.	meters
Vertical play	Ratio of vertical passes (toward opponent's goal) to horizontal passes (lateral movement).	ratio
Throw-in length	Average distance between throw-in location and receiving player position.	meters
Offsides	Total number of offside infractions committed by the team.	count
Pass center of mass (x)	Mean horizontal (longitudinal) coordinate of all successful pass starting locations; proxy for average attacking depth.	meters
Additional metrics computed		
Total events	Total number of all recorded events (passes, shots, fouls, etc.) by the team.	count
Number of shots	Total number of shots taken by the team.	count
Spatial entropy	Normalized average distance between consecutive successful pass locations.	—
High/Low pass accuracy	Accuracy of aerial and bouncing passes, respectively.	%
Max/Avg pass length	Maximum and average length of completed passes.	meters
Number of substitutions	Number of player substitutions made by the team.	count
Shot accuracy	Percentage of shots on target (blocked, saved, or goal).	%
Average shot xG	Average expected goals value per shot attempt.	xG
Shot execution xG uplift	Average difference between post-shot xG and pre-shot xG.	xG
Average shot angle	Average angle subtended by the two goalposts from shot location.	degrees
Pass center of mass (y)	Mean lateral (transverse) coordinate of successful pass starting locations.	meters
Possession	Percentage of match time during which the team controlled the ball.	%
Max PPP / Max PBS	Maximum passes per possession / before shot in any single sequence.	passes
Effective time	Duration of active play per team, excluding stoppages.	seconds
Goal difference	Total goals scored minus goals conceded.	count

Table 2. Supplementary Table S2. Statistical performance metrics computed from match event data. Metrics are divided into those reported in the main analysis (top section) and additional measures computed for exploratory analysis (bottom section).

Metric	Description	Level
	Reported in article	
Outreach (mean)	Spatially weighted dispersion of passes; sum of pass counts multiplied by Euclidean distance between connected regions, normalized by total strength. Higher values indicate longer-range ball circulation.	Network
Maximum eigenvalue	Principal eigenvalue of weighted adjacency matrix; reflects overall connectivity and hierarchical structure. Higher values suggest more centralized passing patterns.	Network
Average shortest path	Mean minimum number of steps to connect any two regions (using inverse weights as distances). Lower values indicate more direct ball progression.	Network
	Additional metrics computed	
In-strength	Total weight of incoming edges to a node; represents number of passes received by a pitch position.	Node (aggregated)
Out-strength	Total weight of outgoing edges from a node; represents number of passes originated from a pitch position.	Node (aggregated)
Betweenness centrality	Fraction of shortest paths passing through a node; identifies most commonly traversed pitch regions.	Node (aggregated)
Eigenvector centrality	Element of principal eigenvector; represents importance of pitch position within passing network.	Node (aggregated)
In/Out-closeness	Average distance from/to all other nodes; indicates accessibility of a pitch position for receiving/distributing the ball.	Node (aggregated)
Total strength	Sum of all edge weights; equivalent to total number of successful passes.	Network
Isolated nodes	Number of pitch regions with no incoming or outgoing passes.	Network
Self-loops	Passes that start and end in the same region (mean weight, max weight, count).	Network
Degree assortativity	Correlation between node degrees; indicates whether highly connected regions tend to connect to other highly connected regions (computed for in-in, out-out, in-out, out-in combinations).	Network

Table 3. Supplementary Table S3. Network metrics computed from pitch-passing networks. Metrics are divided into those reported in the main analysis (top section) and additional measures computed for exploratory analysis (bottom section). Node-level measures were aggregated as mean and standard deviation across all 50 pitch regions.

Network metrics

In Table 3, we list the network measures analyzed in this work.

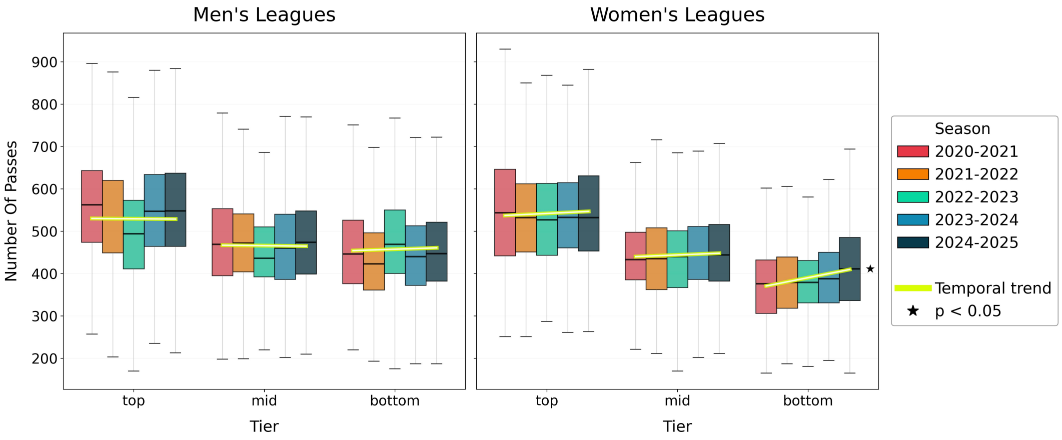

Figure 2. Supplementary Figure S2. Total number of passes by tier for men's (left) and women's (right) football across five seasons. Top teams maintain the highest passing volumes across all seasons, with women's bottom-tier teams showing the only significant temporal increase.

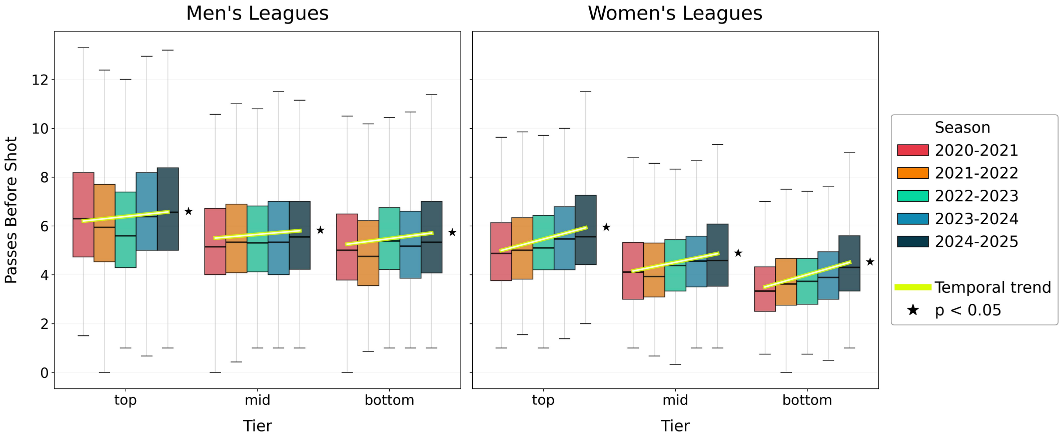

Figure 3. Supplementary Figure S3. Passes before shot by tier for men's (left) and women's (right) football. All tiers exhibit increasing trends, with women's football showing significant growth across all competitive levels (all $p < 0.05$).

Additional performance metrics

The following sections present detailed temporal and hierarchical patterns for supplementary performance indicators not included in the main manuscript. These metrics provide additional context for understanding gameplay changes across men's and women's football. Each subsection examines tier-specific trends and gender comparisons, complementing the core findings reported in the article.

Passing volume and shot buildup

Figure 2 presents the total number of passes completed by teams across tiers and seasons. Top-tier teams consistently complete the highest pass volumes (men: $\sim$541, women: $\sim$543), followed by mid-tier ($\sim$470 and $\sim$444) and bottom-tier teams ($\sim$451 and $\sim$391). Women's bottom-tier teams show a notable increasing pattern over time (slope = 8.69, $p = 0.04$), suggesting potential tactical development at lower competitive levels, though this trend is not observed in other tiers or in men's football.

Passes before shot (PBS), quantifying buildup length prior to goal attempts, exhibits the expected performance hierarchy with top teams recording the highest values (Figure 3). All tiers in both genders show increasing patterns over the five-year period, with women's football demonstrating particularly consistent growth across all competitive levels. This temporal trend complements the increases observed in passes per possession, collectively indicating a shift toward more deliberate attacking sequences.

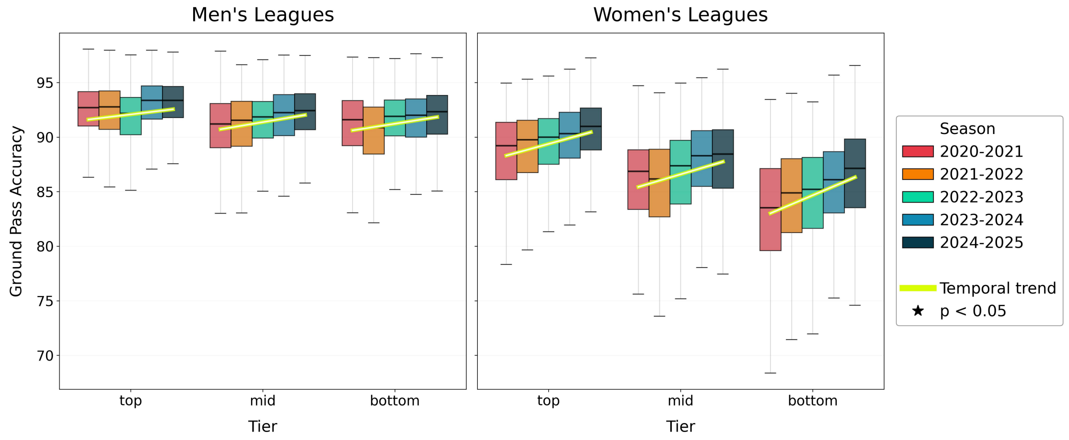

Figure 4. Supplementary Figure S4. Ground pass accuracy by tier for men's (left) and women's (right) football. Women's competitions show stronger temporal increases than men's, with significant trends in top, mid (men only), and bottom tiers.

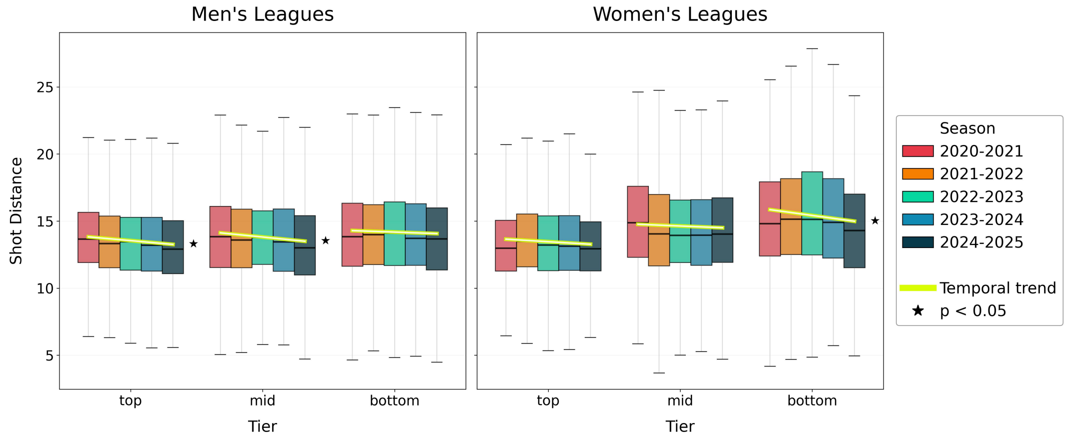

Figure 5. Supplementary Figure S5. Average shot distance to goal by tier for men's (left) and women's (right) football. Men's top and mid-tier teams show significant declining trends, indicating progressively closer shooting positions over time.

Passing precision and shooting behavior

Ground pass accuracy—the completion rate of passes made at ground level—reveals systematic gender differences and temporal evolution (Figure 4). Men's leagues average 90-95% accuracy with modest increases over time, particularly in mid-tier teams. Women's leagues average 85-90% with stronger positive trends, especially pronounced in top and bottom tiers. The gender gap ranges from approximately 3% (top teams) to 8% (bottom teams), consistent with overall passing accuracy patterns.

Shot distance exhibits a consistent declining trend across most tiers in men's football (Figure 5), with top and mid-tier teams showing significant decreases of approximately 0.14-0.15m per year. Women's football shows similar declining patterns, though not reaching statistical significance in most cases. These trends suggest teams are working the ball closer to goal before shooting, aligning with the observed increases in passes before shot and the shorter average pass lengths.

Throw-in length shows modest tier-based differences in both men's and women's leagues, with no consistent ranking pattern across top, mid, and bottom tiers (Figure 6). Temporal trends diverge sharply by gender. Men's leagues exhibit stable or increasing throw-in distances, with mid-tier teams showing a statistically significant positive trend. Women's leagues display the opposite pattern, with decreasing distances and significant negative trends across all tiers, suggesting gender-divergent tactical conventions around restart play.

Network structural properties

Average shortest path length quantifies the efficiency of ball circulation through the network (Figure 7). Top teams maintain the shortest paths (3.2-3.4), enabling rapid ball progression to any pitch region. Men's top-tier teams show a slight increasing pattern, while women's football exhibits relatively stable values across all tiers. The modest changes in this metric, combined with declining network outreach, suggest that teams are maintaining circulation efficiency while concentrating spatial distribution.

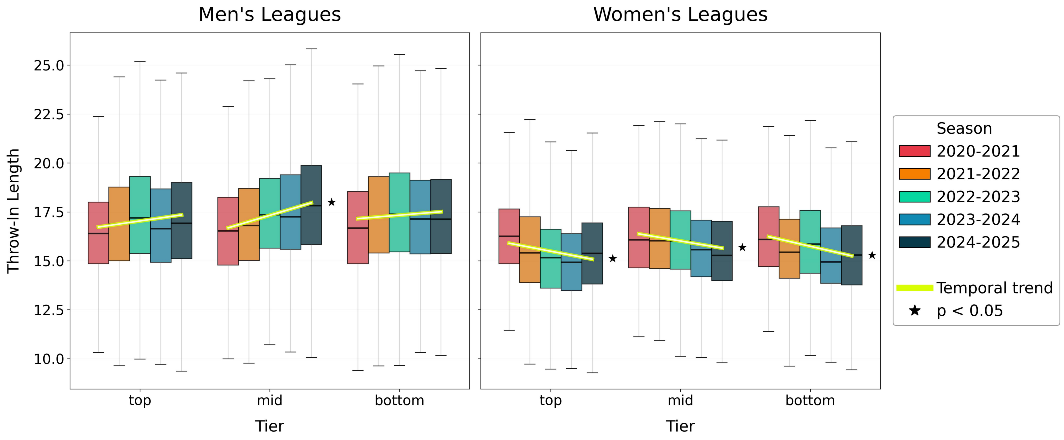

Figure 6. Supplementary Figure S6. Average throw-in length by tier for men's (left) and women's (right) football. Men's leagues show stable or increasing distances, with mid-tier teams exhibiting a significant positive trend. Women's leagues display the opposite pattern, with decreasing distances and significant negative trends across all tiers.

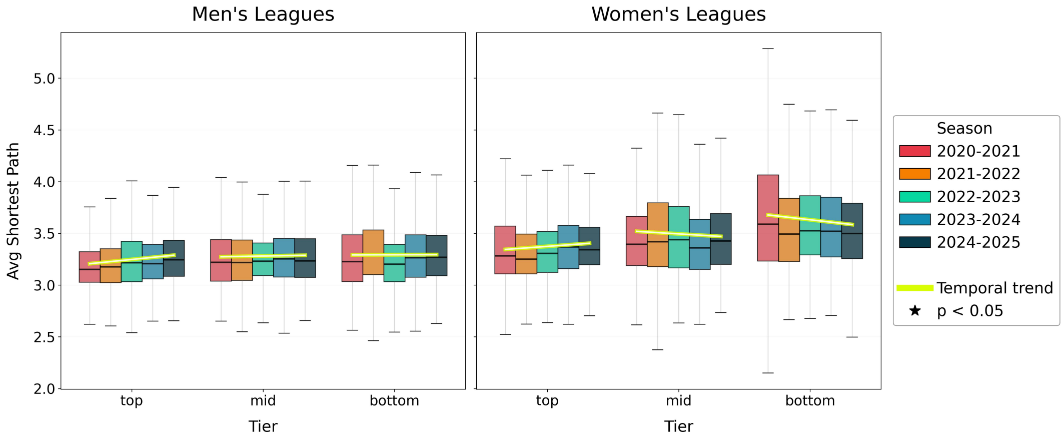

Figure 7. Supplementary Figure S7. Average shortest path length in pitch-passing networks by tier for men's (left) and women's (right) football. Lower values indicate more efficient ball circulation. Men's top teams show a modest increasing pattern, though not statistically significant.

Metric	Men			Women		
	Top	Mid	Bottom	Top	Mid	Bottom
Number of passes	-1.20	-0.77	1.67	1.37	1.85	8.69*
Passes per possession	0.15	0.13*	0.13	0.18*	0.14*	0.19*
Pass accuracy (%)	0.31	0.38	0.43	0.91*	0.67	1.55*
Ground pass accuracy (%)	0.22	0.33*	0.30	0.54*	0.52	0.84*
Passes before shot	0.08	0.07*	0.11	0.23*	0.17*	0.24*
Passes under pressure	3.52*	2.75*	2.71*	3.79*	2.83*	3.68*
Shot distance (m)	-0.14*	-0.15*	-0.05	-0.07	-0.09	-0.16
Max eigenvalue	0.03	-0.02	0.05	0.34	0.12	0.36*
Avg shortest path	0.02*	0.00	0.00	0.01	-0.01	-0.03
Network outreach	-0.18*	-0.06*	-0.15*	-0.36*	-0.25*	-0.40*
Vertical play	0.01	0.01	0.01	0.02*	0.02	0.04*
Throw-in length (m)	0.16	0.32*	0.09	-0.25	-0.17*	-0.26

Table 4. Supplementary Table S4. Temporal trends in match performance metrics (2020-2025). Values represent annual slope from linear regression. Significance assessed at $p < 0.05$ (two-tailed, uncorrected). For Benjamini–Hochberg corrected p-values, see Supplementary Table S9.

Metric	Men				Women			
	Top	Mid	Bottom	F	Top	Mid	Bottom	F
Number of passes	541.1	469.6	451.4	32.6*	542.9	444.1	391.1	293.6*
Passes per possession	6.2	5.4	5.2	14.7*	5.5	4.5	3.9	37.8*
Pass accuracy (%)	81.9	78.4	77.4	21.2*	78.2	72.6	68.7	33.3*
Ground pass acc. (%)	92.3	91.4	91.2	5.4*	89.4	86.6	84.6	24.0*
Passes before shot	6.5	5.7	5.4	25.0*	5.5	4.5	4.0	22.7*
Passes under pressure	55.0	48.4	47.4	308.3*	60.3	50.2	44.4	306.7*
Shot distance (m)	13.5	13.8	14.1	9.3*	13.4	14.7	15.4	56.4*
Max eigenvalue	16.4	13.6	13.1	26.4*	15.8	12.5	10.7	106.6*
Avg shortest path	3.2	3.3	3.3	6.4*	3.4	3.5	3.6	43.2*
Network outreach	21.2	22.0	22.1	669.6*	20.4	21.2	21.4	255.4*
Vertical play	1.2	1.1	1.0	50.2*	1.0	0.9	0.8	32.5*
Throw-in length (m)	17.0	17.3	17.3	0.9	15.5	16.0	15.8	1.4

Table 5. Supplementary Table S5. Within-tier comparison of match performance metrics. Values represent tier means; F = ANOVA F-statistic. *p < 0.05.

Statistical significance analysis

The following tables provide comprehensive statistical summaries of all analyses presented throughout this study. Table 4 reports temporal trend coefficients for each metric across tiers and genders. Table 5 presents within-tier comparisons, demonstrating systematic performance hierarchies. Table 6 quantifies gender differences within each competitive tier. Significance levels are indicated by asterisks (*p < 0.05).

Offsides by country

Offside infractions provide insight into tactical risk-taking and attacking positioning. Table 7 presents offside counts by country, revealing that Spain exhibits significantly elevated offside rates compared to all other leagues in both men's (+20%) and women's football (+21%). This pattern remained stable across the five-year period with no significant temporal trends observed.

Spatial positioning: center of mass

The x-coordinate of passing center of mass quantifies average attacking depth, indicating how high up the pitch teams position their buildup play. Table 8 reveals that men's teams consistently position 6.5% higher upfield than women's teams. Notably, Serie A Women exhibited a strong temporal increase in attacking positioning (+0.82 m/year), while other competitions showed minimal or opposite trends.

Multiple comparison correction

The analyses reported in this study involve a large number of statistical tests across metrics, tiers, genders, and seasons. To assess the robustness of our findings to multiple comparisons, we applied Benjamini–Hochberg (BH/FDR) correction to all

Metric	Top			Mid			Bottom		
	M	W	Δ%	M	W	Δ%	M	W	Δ%
Number of passes	541.1	542.9	-0.3	469.6	444.1	+5.7*	451.4	391.1	+15.4*
Passes per poss.	6.2	5.5	+12.8*	5.4	4.5	+20.9*	5.2	3.9	+31.5*
Pass accuracy (%)	81.9	78.2	+4.8*	78.4	72.6	+7.9*	77.4	68.7	+12.6*
Ground pass acc. (%)	92.3	89.4	+3.3*	91.4	86.6	+5.4*	91.2	84.6	+7.7*
Passes before shot	6.5	5.5	+19.2*	5.7	4.5	+25.2*	5.4	4.0	+35.1*
Passes under pressure	55.0	60.3	-8.9*	48.4	50.2	-3.5*	47.4	44.4	+6.7*
Shot distance (m)	13.5	13.4	+0.6	13.8	14.7	-5.9*	14.1	15.4	-8.1*
Max eigenvalue	16.4	15.8	+3.5	13.6	12.5	+9.6*	13.1	10.7	+22.6*
Avg shortest path	3.2	3.4	-4.1*	3.3	3.5	-6.0*	3.3	3.6	-9.1*
Network outreach	21.2	20.4	+4.0*	22.0	21.2	+3.9*	22.1	21.4	+3.0*
Vertical play	1.2	1.0	+14.1*	1.1	0.9	+20.3*	1.0	0.8	+25.1*
Throw-in length (m)	17.0	15.5	+9.7*	17.3	16.0	+8.2*	17.3	15.8	+9.9*

Table 6. Supplementary Table S6. Gender comparison of match performance metrics by tier. M = Men, W = Women, Δ% = percent difference (positive = men higher). *p < 0.05.

Country	Men	Women	Combined
Spain	2.06	2.54	2.22***
Germany	1.85	2.27	1.98
Italy	1.67	2.26	1.81
England	1.72	1.95	1.78
USA	1.65	1.93	1.71
**Others (pooled)**	**1.71**	**2.10**	**1.81**

Table 7. Supplementary Table S7. Offsides per match by country (2020-2025). Spain shows significantly higher offsides than all other countries pooled in both men's (2.06 vs 1.71, t=16.60, p<0.0001) and women's football (2.54 vs 2.10, p<0.0001), representing a +20% and +21% increase respectively. No significant temporal trends were observed (all p>0.05). ***p < 0.0001 vs pooled others.

tests, organised by metric family. Each family comprises the full set of tests conducted for a given metric: six temporal trend tests (linear regression per gender × tier), two one-way ANOVAs (one per gender), and three gender comparisons by tier, for a total of 11 tests per family. For offsides and pass center of mass, corrections are applied within their respective families (8 and 7 tests, respectively). Table 9 reports raw and BH-corrected p-values for all tests. All findings reported in the main text remain significant under BH correction; minor exceptions occur for supplementary temporal trends reported in Table S4, where four comparisons lose significance after correction.

Table 9. Supplementary Table S9. Multiple comparison correction for all statistical tests. All p-values reported in Tables S4–S8, organised by metric family. Benjamini–Hochberg (BH/FDR) correction is applied within each family. Stars: $^{*}p < 0.05$; $^{**}p < 0.01$; $^{***}p < 0.001$; ns = not significant.

Metric	Test	Comparison	p (raw)	p (BH)
Passes per possession	Temporal	men | bottom	0.2167	0.2384
	Temporal	men | mid	0.0084**	0.0184*
	Temporal	men | top	0.2867	0.2867
	Temporal	women | bottom	0.0143*	0.0224*
	Temporal	women | mid	0.0322*	0.0393*
	Temporal	women | top	0.0232*	0.0320*
	ANOVA	men: top/mid/bot	0.0006***	0.0016**
	ANOVA	women: top/mid/bot	<0.0001***	0.0001***
	Gender	bottom	0.0002***	0.0009***
	Gender	mid	0.0002***	0.0009***
	Gender	top	0.0135*	0.0224*
	Temporal	men | bottom	0.3207	0.3528

(continued on next page)

Pass accuracy

(continued from previous page)

Metric	Test	Comparison	p (raw)	p (BH)
	Temporal	men | mid	0.0989	0.1359
	Temporal	men | top	0.5603	0.5603
	Temporal	women | bottom	0.0040**	0.0063**
	Temporal	women | mid	0.1135	0.1387
	Temporal	women | top	0.0003***	0.0006***
	ANOVA	men: top/mid/bot	0.0001***	0.0003***
	ANOVA	women: top/mid/bot	<0.0001***	0.0001***
	Gender	bottom	0.0001***	0.0003***
	Gender	mid	<0.0001***	0.0002***
	Gender	top	0.0032**	0.0058**
Passes under pressure	Temporal	men | bottom	<0.0001***	<0.0001***
	Temporal	men | mid	<0.0001***	<0.0001***
	Temporal	men | top	<0.0001***	<0.0001***
	Temporal	women | bottom	<0.0001***	<0.0001***
	Temporal	women | mid	<0.0001***	<0.0001***
	Temporal	women | top	<0.0001***	<0.0001***
	ANOVA	men: top/mid/bot	<0.0001***	<0.0001***
	ANOVA	women: top/mid/bot	<0.0001***	<0.0001***
	Gender	bottom	<0.0001***	<0.0001***
	Gender	mid	0.0005***	0.0014**
	Gender	top	<0.0001***	<0.0001***
Shot distance	Temporal	men | bottom	0.3932	0.4325
	Temporal	men | mid	0.0140*	0.0256*
	Temporal	men | top	0.0080**	0.0176*
	Temporal	women | bottom	0.2384	0.3278
	Temporal	women | mid	0.3469	0.4240
	Temporal	women | top	0.1673	0.2630
	ANOVA	men: top/mid/bot	0.0036**	0.0099**
	ANOVA	women: top/mid/bot	<0.0001***	<0.0001***
	Gender	bottom	0.0002***	0.0010**
	Gender	mid	0.0008***	0.0028**
	Gender	top	0.5195	0.5195
Network outreach	Temporal	men | bottom	0.0100**	0.0219*
	Temporal	men | mid	0.3408	0.3748
	Temporal	men | top	0.4729	0.4729
	Temporal	women | bottom	0.0062**	0.0171*
	Temporal	women | mid	0.0790	0.1194
	Temporal	women | top	0.0028**	0.0152*
	ANOVA	men: top/mid/bot	0.0010**	0.0112*
	ANOVA	women: top/mid/bot	0.0208*	0.0381*
	Gender	bottom	0.0868	0.1194
	Gender	mid	0.0050**	0.0171*
	Gender	top	0.1899	0.2321
Max. eigenvalue	Temporal	men | bottom	0.7516	0.9186
	Temporal	men | mid	0.8805	0.9462
	Temporal	men | top	0.9462	0.9462
	Temporal	women | bottom	0.0387*	0.0852
	Temporal	women | mid	0.3722	0.5430
	Temporal	women | top	0.0883	0.1618
	ANOVA	men: top/mid/bot	<0.0001***	0.0002***
	ANOVA	women: top/mid/bot	<0.0001***	<0.0001***
	Gender	bottom	0.0001***	0.0004***

(continued on next page)

(continued from previous page)

Metric	Test	Comparison	p (raw)	p (BH)
	Gender	mid	0.0011**	0.0031**
	Gender	top	0.3949	0.5430
Avg. shortest path	Temporal	men | bottom	0.9727	0.9727
	Temporal	men | mid	0.3789	0.4631
	Temporal	men | top	0.0483*	0.0885
	Temporal	women | bottom	0.0834	0.1310
	Temporal	women | mid	0.5408	0.5949
	Temporal	women | top	0.2976	0.4092
	ANOVA	men: top/mid/bot	0.0129*	0.0284*
	ANOVA	women: top/mid/bot	<0.0001***	<0.0001***
	Gender	bottom	<0.0001***	<0.0001***
	Gender	mid	<0.0001***	<0.0001***
	Gender	top	0.0002***	0.0006***
Number of passes	Temporal	men | bottom	0.7814	0.9095
	Temporal	men | mid	0.8326	0.9095
	Temporal	men | top	0.9095	0.9095
	Temporal	women | bottom	0.0376*	0.0827
	Temporal	women | mid	0.2462	0.4514
	Temporal	women | top	0.6117	0.9095
	ANOVA	men: top/mid/bot	<0.0001***	0.0001***
	ANOVA	women: top/mid/bot	<0.0001***	<0.0001***
	Gender	bottom	0.0002***	0.0009***
	Gender	mid	0.0006***	0.0015**
	Gender	top	0.8903	0.9095
Passes before shot	Temporal	men | bottom	0.1966	0.2163
	Temporal	men | mid	0.0080**	0.0124*
	Temporal	men | top	0.5274	0.5274
	Temporal	women | bottom	0.0095**	0.0124*
	Temporal	women | mid	0.0102*	0.0124*
	Temporal	women | top	0.0005***	0.0011**
	ANOVA	men: top/mid/bot	<0.0001***	0.0003***
	ANOVA	women: top/mid/bot	0.0001***	0.0003***
	Gender	bottom	0.0002***	0.0004***
	Gender	mid	<0.0001***	0.0003***
	Gender	top	0.0015**	0.0027**
Throw-in length	Temporal	men | bottom	0.3962	0.4358
	Temporal	men | mid	0.0018**	0.0049**
	Temporal	men | top	0.2248	0.3091
	Temporal	women | bottom	0.0764	0.1401
	Temporal	women | mid	0.0310*	0.0683
	Temporal	women | top	0.1239	0.1947
	ANOVA	men: top/mid/bot	0.4512	0.4512
	ANOVA	women: top/mid/bot	0.2750	0.3361
	Gender	bottom	0.0003***	0.0034**
	Gender	mid	0.0011**	0.0041**
	Gender	top	0.0008***	0.0041**
Vertical play	Temporal	men | bottom	0.1848	0.2033
	Temporal	men | mid	0.0590	0.0811
	Temporal	men | top	0.4057	0.4057
	Temporal	women | bottom	0.0187*	0.0293*
	Temporal	women | mid	0.1079	0.1319
	Temporal	women | top	0.0148*	0.0271*

(continued on next page)

(continued from previous page)

Metric	Test	Comparison	p (raw)	p (BH)
	ANOVA	men: top/mid/bot	$<0.0001^{***}$	$<0.0001^{***}$
	ANOVA	women: top/mid/bot	$<0.0001^{***}$	0.0001^{***}
	Gender	bottom	0.0001^{***}	0.0003^{***}
	Gender	mid	$<0.0001^{***}$	$<0.0001^{***}$
	Gender	top	0.0001^{***}	0.0002^{***}
Offsides	t-test	Spain vs Others (all)	$<0.0001^{***}$	$<0.0001^{***}$
	ANOVA	all countries	$<0.0001^{***}$	$<0.0001^{***}$
	t-test	Spain vs England	$<0.0001^{***}$	$<0.0001^{***}$
	t-test	Spain vs Germany	$<0.0001^{***}$	$<0.0001^{***}$
	t-test	Spain vs Italy	$<0.0001^{***}$	$<0.0001^{***}$
	t-test	Spain vs USA	$<0.0001^{***}$	$<0.0001^{***}$
	t-test	Spain vs Others (men)	$<0.0001^{***}$	$<0.0001^{***}$
	t-test	Spain vs Others (women)	$<0.0001^{***}$	$<0.0001^{***}$
Pass CoM (x)	Temporal	All Men	$<0.0001^{***}$	$<0.0001^{***}$
	Temporal	Women excl. Italy	0.0300^{*}	0.0350^{*}
	Temporal	Serie A Women	$<0.0001^{***}$	$<0.0001^{***}$
	ANOVA	all groups	$<0.0001^{***}$	$<0.0001^{***}$
	t-test	Men vs Women excl. Italy	$<0.0001^{***}$	$<0.0001^{***}$
	t-test	Men vs Serie A Women	$<0.0001^{***}$	$<0.0001^{***}$
	t-test	Women excl. Italy vs Serie A	0.8854	0.8854

Effective playing time

Proxy for effective time

Effective playing time — the duration of active play excluding stoppages such as throw-ins, fouls, VAR reviews, and injury time — is not directly recoverable from event-based data. StatsBomb event records provide timestamps for individual on-pitch actions, but stoppages are not systematically marked as discrete events, making reliable reconstruction of ball-in-play duration error-prone and potentially biased across leagues and seasons.

As a proxy for effective playing time, we use the total number of passes completed per match. Passing volume scales directly with active play duration: more time in play affords more opportunities to circulate the ball, while stoppages interrupt passing sequences. Supplementary Figure 8 shows the distribution of total passes per match by season and competition for men's and women's football. Passing volume is broadly stable across seasons within each league, with no systematic temporal drift that would confound our analyses. Across competitions, men's leagues show modestly higher passing volumes than women's leagues, consistent with known differences in ball-in-play duration [51,52,53]; however, within-gender differences across countries are small relative to the between-gender gap, suggesting that effective time does not vary substantially across the leagues analyzed here.

Metrics affected by effective playing time

Among the performance indicators analyzed in this study, only count-based metrics — those expressed as absolute numbers of events per match rather than ratios or network-structural properties — are directly susceptible to effective time variation. Specifically:

- **Passes under pressure**: an absolute count of completed passes made under defensive pressure. A team playing more effective time will accumulate more such events irrespective of tactical intensity.
- **Offsides**: an absolute count of offside infractions per match, which scales with the number of attacking actions and hence with effective time.

All other metrics reported in the main analysis are unaffected by effective time variation for the following reasons. Ratio-based metrics (pass accuracy, ground pass accuracy, passes per possession, vertical play) are normalised by definition and therefore invariant to match duration. Spatial metrics (pass center of mass, shot distance, throw-in length) measure positional

Group	Mean (m)	Temporal Trend (m/year)	Significance
All Men	55.71	−0.12	***
All Women (excl. Italy)	52.30	+0.15	*
**Serie A Women**	**52.33**	**+0.82**	***

Table 8. Supplementary Table S8. Center of mass (x-coordinate) by group (2020-2025). Serie A Women show a strong temporal increase in attacking positioning (+0.82 m/year, p<0.0001), while other groups exhibit minimal or opposite trends (All Men: −0.12 m/year, p<0.0001; Women excl. Italy: +0.15 m/year, p=0.03). Men's teams position significantly higher upfield than women's (+6.5%, p<0.0001), but no difference exists between women's groups (p=0.89). *p < 0.05, ***p < 0.0001.

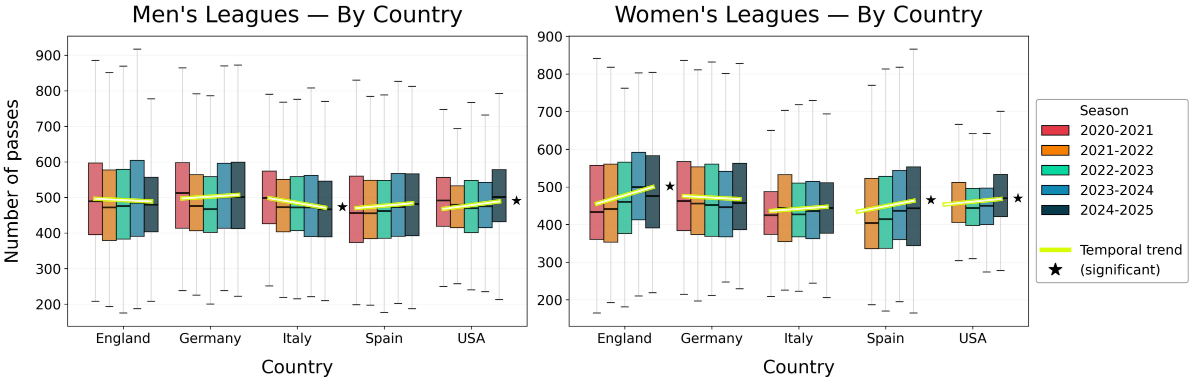

Figure 8. Supplementary Figure S8. Total number of passes per match by country for men's (left) and women's (right) football across five seasons (2020–2025). Passing volume is used as a proxy for effective playing time. Within-gender differences across countries are modest relative to the overall between-gender gap.

quantities independent of event counts. Network-structural metrics (maximum eigenvalue, average shortest path length) are computed on normalized networks and capture topological properties of ball circulation rather than volume. Network outreach is also computed on strength-normalised networks; while the spatial weighting could in principle be influenced by changes in the spatial distribution of passes driven by effective time, this represents a second-order effect that does not alter the directional interpretation of the observed trends.

Normalised metrics

To verify that the main findings for passes under pressure and offsides are robust to effective time variation, we express both metrics as a percentage of total passes per match:

$$\text{Passes under pressure } (\%) = \frac{\text{passes under pressure}}{\text{total passes}} \times 100 \tag{1}$$

$$\text{Offsides } (\%) = \frac{\text{offsides}}{\text{total passes}} \times 100 \tag{2}$$

Supplementary Figures 9 and 10 show the distributions of these normalised metrics by season, country, and tier. The temporal trends in passes under pressure remain significant across all tiers and genders after normalisation (all $p < 0.0001$, Supplementary Table S10), suggesting that the observed intensification of defensive pressure is consistent with a genuine tactical shift rather than an artifact of increasing effective playing time. Notably, the rate of increase is symmetric across genders (≈ 0.6 %/year for both men and women), suggesting a sport-wide phenomenon. The Spain effect in offsides is also preserved after normalisation (Spain = 0.52%, others = 0.38%, $t = 34.58$, $p < 0.0001$), with Spain showing a 35% higher offside rate relative to other leagues — a larger relative difference than observed for the unnormalised metric (+22%).

Statistical validation of normalised metrics

Table 10 reports the full statistical results for the normalised metrics, including Benjamini–Hochberg corrected p-values.

Metric	Test	Comparison	p (raw)	p (BH)
Passes under pressure (%)	Temporal	men | bottom	$<0.0001^{***}$	$<0.0001^{***}$
	Temporal	men | mid	$<0.0001^{***}$	$<0.0001^{***}$
	Temporal	men | top	$<0.0001^{***}$	$<0.0001^{***}$
	Temporal	women | bottom	$<0.0001^{***}$	$<0.0001^{***}$
	Temporal	women | mid	$<0.0001^{***}$	$<0.0001^{***}$
	Temporal	women | top	$<0.0001^{***}$	$<0.0001^{***}$
	ANOVA	men: top/mid/bot	0.9913	0.9913
	ANOVA	women: top/mid/bot	0.4252	0.4723
	Gender	bottom	$<0.0001^{***}$	$<0.0001^{***}$
	Gender	mid	$<0.0001^{***}$	$<0.0001^{***}$
	Gender	top	$<0.0001^{***}$	$<0.0001^{***}$
Offsides (%)	t-test	Spain vs Others (all)	$<0.0001^{***}$	$<0.0001^{***}$
	ANOVA	all countries	$<0.0001^{***}$	$<0.0001^{***}$
	t-test	Spain vs England	$<0.0001^{***}$	$<0.0001^{***}$
	t-test	Spain vs Germany	$<0.0001^{***}$	$<0.0001^{***}$
	t-test	Spain vs Italy	$<0.0001^{***}$	$<0.0001^{***}$
	t-test	Spain vs USA	$<0.0001^{***}$	$<0.0001^{***}$
	t-test	Spain vs Others (men)	$<0.0001^{***}$	$<0.0001^{***}$
	t-test	Spain vs Others (women)	$<0.0001^{***}$	$<0.0001^{***}$

Table 10. Supplementary Table S10. Statistical tests for normalised metrics. Passes under pressure (%) and offsides (%) are expressed as a fraction of total passes per match. Benjamini–Hochberg (BH/FDR) correction applied within each metric family. Stars: $^{*}p < 0.05$; $^{**}p < 0.01$; $^{***}p < 0.001$.

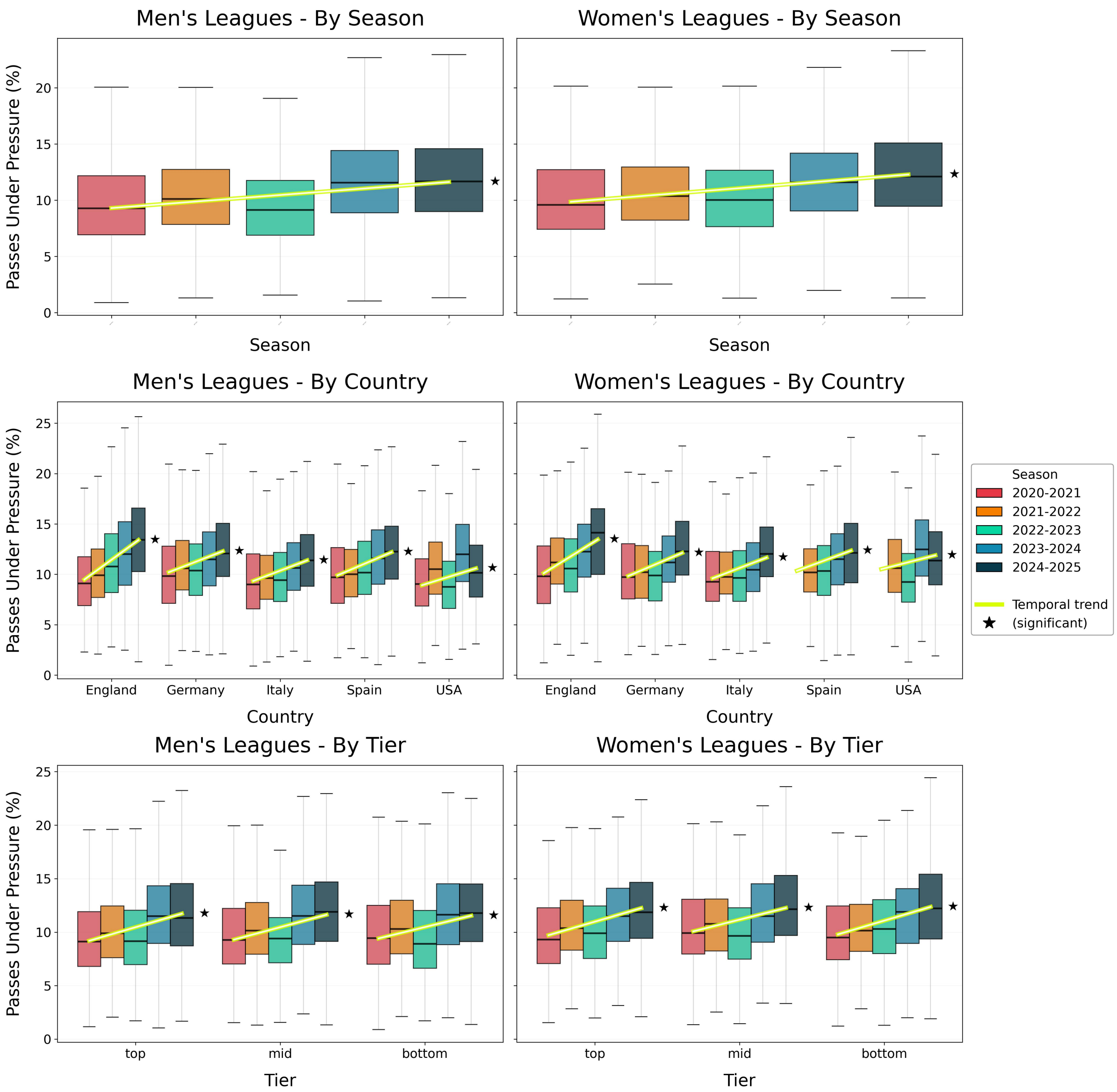

Figure 9. Supplementary Figure S9. Passes completed under pressure as a percentage of total passes per match, shown by season, country, and tier for men's (left) and women's (right) football. Temporal trends remain significant across all groups after normalisation (all $p < 0.0001$), confirming that the intensification of defensive pressure reflects a genuine tactical shift independent of effective playing time.

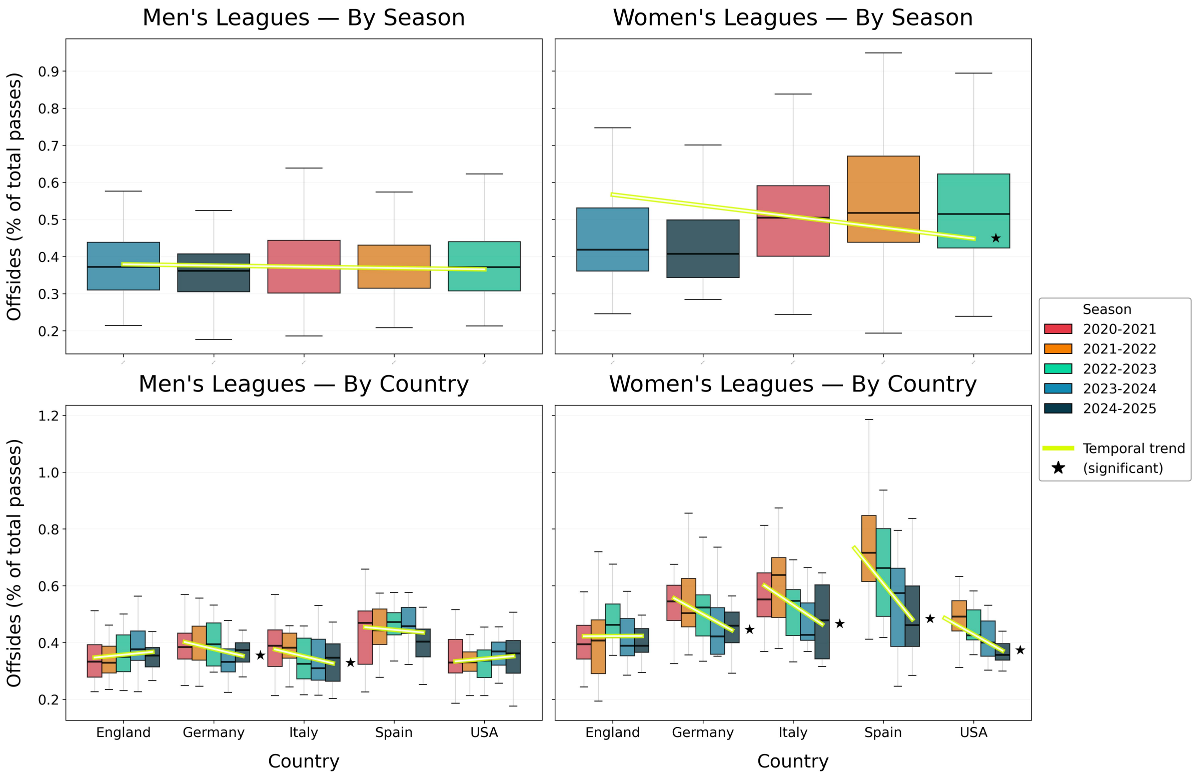

Figure 10. **Supplementary Figure S10.** Offsides as a percentage of total passes per match, shown by season and country for men's (left) and women's (right) football. Spain maintains a consistently higher offside rate than all other countries after normalisation (+35%, $t = 34.58$, $p < 0.0001$), confirming that the country-level difference is independent of effective playing time.
